# Therapeutic approach of natural products that treat osteoporosis by targeting epigenetic modulation

**DOI:** 10.3389/fgene.2023.1182363

**Published:** 2023-05-23

**Authors:** Guokai Zhang, Zhenying Liu, Zihan Li, Bing Zhang, Pengyu Yao, Yun Qiao

**Affiliations:** ^1^ Binzhou Hospital of Traditional Chinese Medicine, Binzhou, China; ^2^ Institute of Chinese Materia Medica, China Academy of Chinese Medical Sciences, Beijing, China; ^3^ The First Affiliated Hospital of Shandong First Medical University Qianfoshan Hospital of Shandong Province, Jinan, China; ^4^ Shandong University of Traditional Chinese Medicine, Jinan, China; ^5^ Shandong Laboratory of Engineering Technology Suzhou Biomedical Engineering and Technology Chinese Academy of Sciences, Jinan, China; ^6^ Jinan Guoke Medical Engineering and Technology Development Company, Jinan, China; ^7^ Qilu Hospital of Shandong University, Jinan, China

**Keywords:** epigenetics, osteoporosis, natural products, mechanism, pathway

## Abstract

Osteoporosis (OP) is a metabolic disease that affects bone, resulting in a progressive decrease in bone mass, quality, and micro-architectural degeneration. Natural products have become popular for managing OP in recent years due to their minimal adverse side effects and suitability for prolonged use compared to chemically synthesized products. These natural products are known to modulate multiple OP-related gene expressions, making epigenetics an important tool for optimal therapeutic development. In this study, we investigated the role of epigenetics in OP and reviewed existing research on using natural products for OP management. Our analysis identified around twenty natural products involved in epigenetics-based OP modulation, and we discussed potential mechanisms. These findings highlight the clinical significance of natural products and their potential as novel anti-OP therapeutics.

## 1 Introduction

Osteoporosis is a common illness that afflicts the elderly population. It is a persistent bone metabolic disease marked with vastly reduced bone mineral density (BMD), bone mass, bone quality, and bone micro-architectural degeneration. Osteoporosis is a dangerous condition due to its high risk of bone fracture. Worldwide, there have been over 8.9 million reported cases of osteoporotic fractures (Salari et al., 2021), and statistics suggest that one in two adult women or one in five adult men experience at least one fragility fracture in their lifetime (Gregson et al., 2022). Fractures can lead to the immobilization of patients and increase the risk of fatal secondary complications, particularly in the elderly.

OP is a life-long condition with no available cure (Gregson et al., 2022). Hence, it is both urgent and necessary to develop a novel and effective therapy for OP (Lin et al., 2022). There has been much interest in developing an alternative form of care for OP patients in recent years. Emerging evidence suggests that several ingredients, compounds, and their combinations protect against bone loss and preserve bone micro-structural integrity (Wang et al., 2017). Several reports demonstrated the highly efficacious nature of multiple natural plants and Chinese herbal medicines, particularly in bone preservation, with minimal undesirable side effects and suitability for long-term usage regarding chemically synthesized drugs (An et al., 2016; Zhou T et al., 2021).

Natural products have emerged as potential candidates for preventing OP in recent years. However, the precise mechanism by which they act remains unknown, leading to limited clinical applications. In response to this challenge, epigenetics has gained increasing attention in OP research due to its ability to study environmental factors and associated genetic lesions (Xu et al., 2021). Using epigenetics in OP research has significantly enhanced our understanding of epigenetic inheritance, leading to improved diagnostics and therapy. Consequently, a renewed interest has been in identifying compounds that can modulate OP-related mechanisms and their potential therapeutic benefits for patients.

## 2 Epigenetics of OP

Multiple factors modulate OP, including genetics. As such, epigenetic inheritance is critical for its pathology. Prior epigenetic investigations offered the latest information on the underlying mechanisms behind the OP-related pathophysiology and potential anti-OP therapy. OP drivers are recognized OP-related genes that potentially modulate DNA/RNA methylation, non-coding RNAs (ncRNAs), histone modifications, nucleosome positioning (NP), and chromatin configuration to bring about the pathophysiological and therapeutic manifestations of OP.

### 2.1 DNA/RNA methylation

DNA methylation is a common epigenetic modulatory mechanism that regulates developmental processes, normal biology, and disease conditions in numerous eukaryotes (Feng and Lou, 2019). Thus far, scientists have identified several forms of DNA methylation in mammals, namely, 5-methylcytosine (5 mC), 5-hydroxymethylcytosine (5hmC), 5-formylcytsine (5fC), 5-carboxycytosine (5caC), DNA N6-methyldeoxyadenosine (6 mA), and N-7 guanine methylation (7-MG) (Thomas et al., 2013; Meng et al., 2015; Liu J et al., 2016). 5 mC is the most commonly modified base in eukaryotic genomes. DNA methyltransferase enzymes typically transfer a methyl group from the cofactor S-Adenosyl-L methionine (SAM) to the 5′ position of the cytosine ring within DNA (Schmitz et al., 2019). Earlier reports revealed that differential promoter methylations control osteoblastic and osteoclastic development and activities (Ehrlich, 2019). In patients with estrogen deficiency, specific DNA methylation is known to alter OP-related gene expression (Xu Z et al., 2022). Menopausal women often experience postmenopausal OP (PMOP) due to a drastic reduction in estrogen levels after menopause (Fischer and Haffner-Luntzer, 2022). Based on a genome-wide investigation of DNA methylation profiles in PMOP patients and healthy postmenopausal counterparts, there were 8973 differentially methylated genes (DMG, 5200 hyper-, and 3773 hypo-methylated genes) at strongly relevant methylation sites (Zhou et al., 2020). Among the broad regulation of essential bone-related genes, DNA methylation is reported to influence the expressions of OPG and RANKL, which may initiate primary OP (Wang P et al., 2018). Moreover, there are reports of other bone factors, namely, bone morphogenetic protein 2, (BMP2) sclerostin, (SOST), cTBC1 Domain Family Member 8 gene (TBC1D8), and others that modulate OP (Visconti et al., 2021). Furthermore, DNA methylation can also regulate drug efficacy. Emerging evidence suggests that baseline CYP2R1 and CYP24A1 DNA methylation levels may be bioindicators of OP patients’ vitamin D response. Meantime, Vitamin D supplementation can also alter DNA methylation levels of the CYP gene family *in vivo* (Zhou et al., 2014). However, there are still gaps in our knowledge of the association between DNA methylation and OP pathology, and additional investigations are warranted to solidify its role in OP.

RNA methylation is still in its early stages of development, in contrast to the well-established knowledge of DNA methylation. Like DNA methylation, RNA methylation refers to modifications that influence cells’ genetic profile and functionality (Mei et al., 2020). To date, there have been 150 reported RNA modifications, among which the N6-methyladenosine (m6A) modification is highly prevalent in eukaryotes (Chen J et al., 2021). This modification plays a crucial role in bone growth and homeostasis by regulating the expression of key genes that control bone cell proliferation, differentiation, and apoptosis. Examples of such genes include *ALP, Runx2, Osterix*, and *VEGF* (Huang et al., 2021). However, there is limited research on the role of RNA methylation in OP. Supplementary Table S1 presents data from recent studies on the outcomes and mechanisms of DNA/RNA methylation in OP, including the types, genes, targets/pathways, methyltransferases, and resulting effects.

### 2.2 ncRNAs

ncRNAs are transcripts with low or no coding potential. Dysregulations in ncRNAs are known to affect both OP occurrence and progression. ncRNAs can be separated into two categories based on their activity: regulatory and housekeeping. Regulatory ncRNAs include microRNAs (miRNAs), long non-coding RNAs (lncRNAs), small interfering RNA (siRNA), piwi-interacting RNA (piRNA), enhancer RNA (eRNA), and circular RNA (circRNA). In contrast, housekeeping ncRNAs include transfer RNAs (tRNA), ribosomal RNAs (rRNA), small nuclear RNA (snRNA), telomerase RNA (TERC), and small nucleolar RNAs (snoRNAs). miRNAs are small ncRNAs that suppress mRNA stability and translation, thereby controlling gene expression and cellular activity. Regarding bone, miRNAs are reported to modulate bone-forming osteoblast- and bone-resorbing osteoclast-related genes (Ko et al., 2020). An extensive miRNA profile study identified 331 miRNAs as significant in PMOP patients (Li Y, et al., 2020), suggesting that the OP pathology may be influenced by miRNAs (De Martinis et al., 2020). Long non-coding RNAs are molecules longer than 200 nucleotides that do not encode proteins. Numerous studies have established a strong association between lncRNAs and OP regulation. For example, Nron, a lncRNA, has been shown to be a negative modulator of bone resorption and is involved in cell proliferation, apoptosis, and inflammatory response within bone tissue (Jin et al., 2021). In another study, RNA sequencing was used to identify differentially regulated mRNAs and lncRNAs in postmenopausal OP women. A total of 185 mRNAs and 51 lncRNAs were found to be differentially regulated in the postmenopausal OP (Fei et al., 2018). However, the molecular mechanisms by which lncRNAs regulate osteoblasts and osteoclasts remain unclear (He and Chen, 2021).

Compared to microRNAs and lncRNAs, circRNA research is still in its infancy. CircRNAs are single strands of circular RNA that serve as transcriptional modulators, microRNA (miR) sponges, and protein templates (Zhou et al., 2020). Emerging reports identified specific circRNAs, circ_28313, circ_0016624, circ_0006393, circ_0076906, and circ_0048211, as essential regulators of bone metabolism, in particular, bone marrow stromal cells (BMSCs) differentiation, proliferation, and apoptosis (Chen W et al., 2021). With increasing ongoing investigations, more critical circRNAs are being identified.

Multiple reports recognize the aforementioned molecules as modulators of osteoblastic and osteoclastic differentiation via several networks. Hence, pathways, miRNAs, lncRNAs, and circRNAs are strategic therapeutic targets or bioindicators of anti-OP diagnosis and therapy (Yang Y et al., 2020). A complete understanding of the intricate roles of the above molecules will likely involve decades of research. Supplementary Table S2 details the effects and mechanisms underlying the ncRNA-mediated regulation of OP, according to recently published studies. These include the types, RNAs, targets/pathways, and functions of molecules.

### 2.3 Histone modifications and others

Another common form of epigenetic control is histone modifications. Histones are central to the nucleosomal subunit, forming an octamer with four core histone proteins (H3, H4, H2A, H2B) wrapped around a 147-base-pair DNA segment (Audia and Campbell, 2016). When stimulated by various factors, histones undergo posttranslational modifications, which alter the three-dimensional configuration of chromosomes, thereby affecting gene transcription. Histone modification often occurs in the following pattern: acetylation, methylation, phosphorylation, sumoylation, ubiquitylation, ADP ribosylation, butyrylation, citrullination, crotonylation, formylation, proline isomerization, propionylation, serotonylation, and dopaminylation (Geng et al., 2021). Among them, acetylation, methylation, ubiquitination, and ADP-ribosylation are intricately linked to OP development and progression (Sun et al., 2022). Supplementary Table S3 lists the outcomes and mechanisms of histone modifications on OP, as evidenced by recently published investigations. These include the types, names, targets/pathways, and functions.

Approximately 3/4 of eukaryotic DNA is condensed into nucleosomes (Neipel et al., 2020). Nucleosomes are building blocks of eukaryotic chromatin, which includes a short DNA stretch spanning 147 base pairs (bp), which encircles about 1–3/4 turns around a cylindrical aggregate of eight histone proteins. The histone octamer location on a DNA sequence is known as NP. Alterations in NP and spacing influence DNA accessibility to modulatory factors and the formation of higher-order chromatin configurations (Baldi, 2019). The NP of DNA also controls the physiological activities of the DNA itself. The histone octamer harbors two copies of H2A, H2B, H3, and H4. In one study, 3 Cbfa motifs, including A, B, and C, were strategically placed within the bone-specific rat osteocalcin (rOC) promoter, and sites B and C were placed alongside a nucleosome in the proximal promoter. The subtle differences within the Cbfa motif arrangement strongly regulated osteocalcin expression and responsiveness to physiologic mediators of bone formation and turnover (Javed et al., 1999). Another study confirmed that the Atg7 deletion aggregates the H3.1 protein in the cytoplasm by disrupting its nuclear transfer in CD11b^+^ and Ly6G^+^ cells (Fang et al., 2020). Other studies mapped out the human genome-wide nucleosome distributions to explore the nucleosome role in bone metabolism. However, the significance of the chromatin configuration in OP progression is yet to be elucidated.

Chromatin structural modification is essential to the modulation of gene expression. Due to advancements *in situ* technology, capturing data on looping, topological domains, larger chromatin compartments, and chromatin-linked diseases is currently feasible (Wang M et al., 2022). Epigenetic modification modulates OP physiology and pathophysiology by regulating chromatin configuration and gene expression. Histone deacetylases (HDACs) are widely known for their unique ability to alter chromatin configuration and affect gene expression. They also contribute to osteogenic regulation and are excellent candidates for bone-related therapeutic targets, including OP (Lee et al., 2011). HDAC9 was reported to regulate genes associated with chronic diseases, namely OP. It does so by altering the chromatin structure at the DNA promotor location or diminishing the transcriptional activity of related transcription factors (Hu S et al., 2020).

## 3 Progress in OP therapy via regulation of epigenetic inheritance of natural products

OP is a complex disease modulated by various factors such as DNA/RNA methylation, ncRNAs, histone modifications, and NP. Natural products have gained significant attention in OP research in recent years due to their potential therapeutic effects. With the development of epigenetics, new mechanisms underlying the therapeutic actions of natural products in OP have emerged, presenting opportunities for developing highly effective OP treatments.

### 3.1 Natural compounds regulate DNA/RNA methylation

DNA/RNA methylation is a potentially ideal target for drug therapy. DNA methylation-based drug inhibition reduces the methylation level of related genes, thereby managing the pathological alterations that occur with OP. More recently, several natural compounds were reported to serve a clinical function by suppressing DNA/RNA methylation. Given this evidence, there are multiple opportunities for potential targets for OP therapy.

#### 3.1.1 Tanshinone IIA

Tanshinone IIA (Tan IIA) is a potent active ingredient in Salvia miltiorrhiza. It possesses remarkable pharmacological activities, such as anti-oxidant, anti-atherosclerosis, antibacterial, anti-inflammatory, and anti-tumor properties (Guo et al., 2020). Tan IIA is a potential anti-OP drug with great significance in OP treatment. A prior investigation suggested that Tan-IIA protects against oxidative stress during osteoblast differentiation in OP mice by modulating the NF-κB axis (Zhu et al., 2018). OP is among the many complications of diabetes. Moreover, both type 1 (T1D) and type 2 (T2D) diabetes mellitus is correlated with an enhanced osteoporotic fracture risk (Schacter and Leslie, 2021). One study revealed the beneficial effects of Tan IIA on the bones of diabetic mice. Mechanistically, Tan IIA diminished ANG II synthesis by suppressing renin activity to protect against OP in diabetic mice (Zhang et al., 2020). Thus, Tan IIA is a potentially robust and efficacious therapy for POMP prevention. Based on another study, Tan IIA strongly attenuates RANKL-induced osteoclastogenesis by abrogating NF-κB, PI3-kinase/AKT, and MAPK axes activation, in addition to the transcription factor NFATc1 stimulation (Cheng et al., 2018). Glucocorticoid-induced OP (GIOP) is another common secondary contributor to OP among adults (Laurent et al., 2022). Tan IIA suppresses glucocorticoids-induced osteoblast apoptosis and OP by inhibiting the Nox4-derived ROS synthesis (Li B et al., 2019). The above studies provided strong evidence that Tan IIA effectively treats OP. However, the associated mechanism requires further exploration. Epigenetics is involved in the internal mechanisms of Tan IIA’s action. One study suggested that Tan IIA modulates phosphoglycerate dehydrogenase (PHGDH) content by inhibiting PHGDH promoter methylation (Wang L et al., 2019).

#### 3.1.2 Astragalus polysaccharide (AP)

AP is a critical bioactive component of *Astragalus membranaceus* var. *mongholicus* (Bunge) P. K. Hsiao. It is also known to possess numerous health benefits, namely, modulation of immune action, anti-aging, anti-tumor, blood sugar reduction, circulating lipid reduction, anti-fibrosis, antibacterial, radiation protection, and antiviral properties (Zheng et al., 2020). Heteropolysaccharides constitute the major components of AP extracts. Several studies have reported that AP possesses strong anti-osteoporotic properties. In one study, AP effectively mitigated oxidative stress-induced OP in ovariectomized rats by regulating the FoxO3a/Wnt2/β-catenin axis (Ou et al., 2019). These findings suggest that AP could be a promising therapeutic candidate for preventing and treating postmenopausal OP. However, the precise mechanism underlying the observed effects of AP is yet to be fully elucidated (Huo and Sun, 2016). Another study revealed that AP reprograms the intestinal gene profile to relieve OP using the gut-bone network. The DNA methylome provides an epigenetic pathway for the APS-driven alteration of gene expression. However, this modification may not be the main modulatory agent for gene expression. Based on a pilot study involving DNA methylome remodeling in AP, AP severely alters the DNA methylome in colonic epithelia with great efficiency. Therefore, this compound has remarkable anti-OP treatment potential (Liu J et al., 2020).

#### 3.1.3 Gossypol

Gossypol, a natural polyphenol extracted from cotton seed, root, and stem, has been extensively studied for its various beneficial properties, including antifertility, antioxidant, anticancer, antiviral, antiparasitic, and antimicrobial activities, as well as plasma cholesterol reduction (Keshmiri-Neghab and Goliaei, 2014). Gossypol has recently been investigated for its potential as an anti-osteoporotic agent. One study demonstrated that gossypol activates the Wnt/β-catenin pathway, promoting bone formation and reducing cell apoptosis (Liang et al., 2018). The Wnt inhibitory factor 1 (WIF1) was also identified as a critical factor for gossypol treatment. One study confirmed that gossypol suppresses WIF1 levels via methylation of the WIF1 promoter (Liang et al., 2019). Collectively, these findings confirm that gossypol is another potential drug for OP therapy.

#### 3.1.4 Sulforaphane

Sulforaphane is an isothiocyanate phytocompound commonly found in sprouts of cruciferous vegetables, and its therapeutic effects on OP have been extensively studied. According to a recent study, sulforaphane has been shown to prevent osteoblast apoptosis induced by Dex by regulating the Nrf2 axis (Lin et al., 2014). Sulforaphane also possesses beneficial effects in terms of acceleration of osteoblastic differentiation, which is potentially mediated through an epigenetic system via promotion of the ten-eleven translocation 1 (Tet1)/Tet2-dependent hydroxymethylation of DNA, which reactivates gene expression (Thaler et al., 2016).

#### 3.1.5 Rhein

Rhein is a natural compound obtained from *Rheum palmatum* L. and has exhibited strong therapeutic potential against OP. Studies have demonstrated its ability to target bones and inhibit osteoclastogenesis (Jiang M et al., 2019). Moreover, it has been found to reverse OP-induced changes in femurs, possibly by mitigating hypermethylation of the Klotho promoter and DNMT1/DNMT3a activity (Zhang Q et al., 2017).

#### 3.1.6 Theaflavin-3,3′-Digallate (TF3)

TF3, a natural product extracted from black tea, has been shown to exhibit a wide range of physiological activities, similar to other natural products. It also has the potential as a medicine for treating and preventing OP. Research has demonstrated that TF3 accelerates osteoblast differentiation, enhances bone mineralization, and increases bone mass while inhibiting osteoclast formation (Ai et al., 2020; Ge et al., 2021). TF3 likely mediates its action through DNA methylation regulated by DNA methyltransferases. DNMT3a is a major enzyme in DNA methylation. TF3 was shown to modulate DNMT3a to prevent bone loss via inhibition of DNMT3a-mediated epigenetic regulation (Nishikawa et al., 2015).

### 3.2 Natural products modulate ncRNAs

Recent studies have shown a significant association between ncRNAs and human diseases, including OP. As a result, modulation of ncRNAs has become a promising approach to managing OP. Compared to other epigenetic alterations, ncRNAs are increasingly considered a preferred target in drug-related studies. Consequently, there has been a surge in studies focusing on ncRNA-based OP therapy.

#### 3.2.1 Resveratrol

Resveratrol is a polyphenol derived from various sources, such as wine, berries, and peanuts (Galiniak et al., 2019). Resveratrol also possesses osteogenic and osteoinductive activities. It modulates bone cell metabolism and bone turnover (Mobasheri and Shakibaei, 2013). ncRNAs regulation is the primary form of resveratrol anti-OP therapy. Estrogen deficiency-induced OP may benefit most from resveratrol owing to its status as a polyphenolic phytoestrogen. Resveratrol effectively prevents OP in ovariectomized rats via the regulation of microRNA-338-3p (Guo et al., 2015). Additionally, resveratrol improves OP by attenuating the NADPH oxidase 4/nuclear factor kappa B axis via increased miR-92b-3p content (Zhang Y et al., 2020). It was previously confirmed that resveratrol accelerates osteogenic differentiation of bone marrow-derived mesenchymal stem cells (BMSC) via the miR-193a/SIRT7 network (Song C Y et al., 2022). Resveratrol also possesses estrogen-like effects, distinguishing it from other natural products in OP research.

#### 3.2.2 Icariin

Icariin powder, an 8-isopentenyl flavonoid glycoside, is derived from Epimedium. Based on the “kidney dominate bone theory,” Epimedium is widely used in traditional Chinese medicine (TCM) clinical treatment for bone disorders. One study confirmed that icariin has robust bone-promoting activity, making it an excellent candidate for postmenopausal OP therapy (Wang et al., 2017). Icariin modulates bone differentiation by elevating miR-335-5p levels (Teng et al., 2022). Furthermore, Icariin restores bone loss and suppresses OP development and progression by reducing miR-34c levels (Liu J et al., 2016).

The etiology behind drug-induced OP remains unknown; however, its adverse effects are widely recognized. Dexamethasone is one such drug that causes severe bone loss. Icariin was shown to ameliorate dexamethasone-induced bone deterioration in an experimental mouse model via microRNA-186-mediated suppression of cathepsin K (Ma Y et al., 2018).

#### 3.2.3 Puerarin

Puerarin, derived from Pueraria, is an isoflavone highly beneficial for bone health. Pueraria has not been mentioned as a drug to treat bone diseases in TCM, but modern pharmacological studies have confirmed Pueraria’s effectiveness in OP, an innovation for natural drug research. Puerarin prevents bone loss and reduces the risk of OP development (Kulczyński et al., 2021). Runt-related transcription factor-2 (Runx2) is essential in osteoblast differentiation. Several studies confirmed that puerarin induces significant osteoblast cell proliferation, differentiation, and mineralization by downregulating TRPM3/miR-204, modulating [Ca^2+^]I and [Ca^2+^]0, and activating Runx2 (Zhan et al., 2017; Zeng et al., 2018).

#### 3.2.4 Artesunate

Artesunate, a semi-synthetic artemisinin derivative of sesquiterpene lactone, is an antimalarial drug used to treat orthopedic diseases like rheumatoid arthritis, osteoarthritis, and OP (Li J et al., 2015; Ma et al., 2019; Ma Y et al., 2022). Artesunate accelerates osteoblast differentiation and alleviates OP via miR-34a/DKK1 axis regulation (Zeng et al., 2020).

#### 3.2.5 Kaempferol

Kaempferol, a ubiquitous polyphenol in fruits and vegetables, has bone-protecting properties, as evidenced in both *in vitro* and *in vivo* experimental models (Wong et al., 2019). Kaempferol regulates the activities of ncRNAs to alleviate OP. It also accelerates the osteogenic differentiation of BMSCs and alleviates OP via the downregulation of miR-10a-3p (Liu H. et al., 2021).

#### 3.2.6 Astragaloside IV

Astragaloside IV, derived from *Astragalus membranaceus*, is a cycloartane-type triterpene glycoside (Zhang J et al., 2019). Astragaloside IV accelerates osteogenic differentiation of BMSCs via the miR-21/NGF/BMP2/Runx2 axis, alleviating OP (Wu et al., 2020).

#### 3.2.7 Curcumin

Curcumin is a polyphenol found in turmeric. Glucocorticoid-induced OP is a common contributor to secondary OP. One study reported that curcumin enhances the bone microstructure of glucocorticoid-induced secondary OP mice by modulating MMP-9 to activate microRNA-365 (Li B et al., 2019).

#### 3.2.8 Oleanolic acid

Oleanolic acid is a pentacyclic triterpenoid derived from plants with strong anti-OP effects. RANKL signaling drives osteoclastogenesis, which is critical for diseases like OP. Oleanolic acid suppresses RANKL-induced osteoclast production via modulation of the ER α/miR-503/RANK axis, thereby treating OP (Xie et al., 2019).

#### 3.2.9 *Morinda officinalis* polysaccharide

Compared to terpenes and polyphenols, the role of polysaccharides in OP therapy-related epigenetics is not well-understood. However, emerging evidence suggests that polysaccharides could be important in OP treatment. *Morinda officinalis* polysaccharide is a prominent active component found *in Morinda officinalis*. Studies have shown that it can modulate bone-lipid differentiation of BMSCs in osteoporotic rats via upregulation of miR-21 and activation of the PI3K/AKT axis, indicating its potential as a therapeutic agent for OP (Wu PY et al., 2022). Further research is necessary to fully understand the epigenetic mechanisms underlying the therapeutic effects of *Morinda officinalis* polysaccharide and other polysaccharides on OP.

#### 3.2.10 *Polygonatum sibiricum* polysaccharide

*Polygonatum sibiricum* polysaccharide is derived from *Polygonatum sibiricum* and has remarkable anti-OP activity. It modulates osteoclast differentiation by regulating miRNA-1224 to treat OP (Li J et al., 2015).

#### 3.2.11 Psoralen

Psoralen is a furancoumarin found within the leguminous herb Psoralea. Psoralen, a traditional Chinese medicine, has been used for various bone diseases, including OP, based on the theory of “kidney dominate bone.” It modulates the osteogenic differentiation of BMSCs by negatively regulating Runx2 via miR-488 targeting (Huang et al., 2019).

#### 3.2.12 Aloin

Aloin is a natural anthraquinone glycoside extracted from aloe vera. MiR-21 is crucial to the aloin-mediated inhibition of osteoclastogenesis, which is key to preventing osteoporotic diseases like OP (Madhyastha et al., 2019).

### 3.3 Natural products modify histones and regulate other epigenetic factors

OP alters multiple epigenetic events across various species. DNA/RNA methylation, ncRNAs, histone modification, and other epigenetics (NP and chromatin configuration) are significant in OP. However, there is limited information available on the associated mechanisms. The aforementioned epigenetic regulations are key to the intervention mechanism of natural products in OP therapy. Some of the research results are discussed below.

#### 3.3.1 Resveratrol

Resveratrol has been demonstrated to have potent therapeutic effects against OP, with epigenetic alterations playing a key role in its mechanism of action. In addition to its ability to alter DNA methylation and ncRNA levels, as discussed earlier, resveratrol has been shown to modulate histone acetylation. Histone acetyltransferase p300 plays a crucial role in altering chromatin configuration via histone acetylation, which influences gene transcription. Studies have revealed that resveratrol-mediated SIRT-1 interactions with p300 regulate RANKL activation of the NF-κB axis, inhibiting osteoclastogenesis in bone-derived cells. Moreover, resveratrol-activated SIRT-1 deacetylase has been shown to induce SIRT-1-p300 complex formation, which inactivates p300 acetyltransferase and reduces NF-κB-p65 acetylation, ultimately suppressing osteoclast formation and activity (Shakibaei et al., 2011).

#### 3.3.2 Puerarin

As mentioned above, puerarin is a potent natural drug against OP. In general, histone acetylase acetylates targets, and HDAC is a prominent histone acetylase. Puerarin reverses abnormal diabetes-induced (HDAC)-1−3 expressions, making it an excellent candidate for diabetic OP prevention via inhibiting the HDAC1/HDAC3 axis (Guo et al., 2019).

Interestingly, not all natural products benefit OP. Some play the opposite role in terms of bone health. Caffeine, for instance, is an alkaloid found in numerous plants. Caffeine during pregnancy is one of the risk factors for OP in adult offspring. Histone methylation is one pathway whereby caffeine promotes OP. Caffeine exposure diminishes histone methylation of fetal hepatic IGF-1. One report suggested that caffeine-induced fetal rat over-exposure to maternal glucocorticoid and histone methylation of hepatic IGF-1 induces skeletal growth retardation, suggesting caffeine accelerates OP development (Tan et al., 2012). Another research revealed that prenatal caffeine exposure continuously downregulates the H3K9ac content of 11β-HSD2 likely through the downregulation of 11β-HSD2 expression in the male offspring (Xiao H et al., 2020).

## 4 Discussion

Epigenetic mechanisms include all inheritable modulatory networks that influence gene expression without affecting DNA sequence. These include DNA/RNA methylation, ncRNAs, histone modification, NP, and chromatin reconfiguration, and these mechanisms are critical for multiple diseases, including OP. Herein, we summarized the recent progress in epigenetics research, particularly in OP pathology. DNA/RNA methylation and other epigenetic modulation influence the bone formation, osteoblast development, osteogenic and osteoclast differentiation, and OP pathogenesis. The significance of DNA methylation in OP is widely recognized; however, research on RNA methylation is lacking. There are over 200 ncRNA alterations in OP development and progression, among which miRNA is the most reported. Relative to the two studies mentioned above on epigenetic alterations, studies on histone modification are scarce. RUNX2 is a member of the RUNX factor family critical for skeletal development. As a significant osteogenic transcription factor, Runx2 is essential in the early stage of osteogenic differentiation, and it is regarded as a differential bioindicator (Shao H et al., 2021). Our literature review revealed that a large proportion of epigenetic modulations, namely, DNA methylation and ncRNAs influence OP via RUNX2, particularly during bone differentiation. Differentiation from osteoclast precursor cells to fully activated multinucleated osteoclasts critically depends on the receptor activator of NF-κB ligand (RANKL) (Rachner et al., 2011), and this, too, is regulated via epigenetic mechanisms. The epigenetic alterations related to bone metabolism are rather complex, and the current research only analyzed reported modulatory targets. Our findings are a stepping stone for future research into natural products and their action in OP therapy.

Natural products have long been used as early medicines against many chronic and metabolic diseases (Yao and Liu, 2022). These products employ a variety of signaling pathways to reduce or inhibit OP. Many plants that provide natural compounds have a long history of use in traditional medicine or as alternative, complementary therapeutic agents. Some ancient theories supported the pharmaceutical application of these plants and inspired modern pharmacological studies, such as “kidney dominate bone.” Modern innovations in life science, such as epigenetics, have opened a new path for studying natural medicines and their active ingredients. Epigenetic research is highly beneficial in discovering novel signaling pathways associated with a certain stimulus. Approximately twenty natural products are known to intervene with OP using epigenetics. Various natural products, namely, Tan IIA, AP, and Resveratrol, are highly efficacious in treating OP; however, ncRNA-based interventions are the most abundant. Nevertheless, not much is known about the associated mechanisms behind their actions. The structure-efficacy relationship of natural products may provide a good reference for developing new drugs and treating diseases. However, natural products that interfere with OP through epigenetic pathways include terpenoids, flavonoids, polyphenols, polysaccharides, and other types. A few studies reported could not provide evidence and opinions for the structure-efficacy relationship of natural products in the treatment of OP. Secondary osteoporosis may be a breakthrough in the study of natural products, and there are reports demonstrating the feasibility of natural products in their treatment *in vivo* and *in vitro*, such as diabetes-induced osteoporosis. Although studies on natural products have provided some insight into epigenetics regulation in treating OP, there is still much to uncover. The genetic intervention basis for the epigenetic regulation of natural products is not yet fully understood, likely due to the limited number of studies analyzed. To develop targeted and effective anti-OP therapy, further research is necessary to explore the epigenetic control of natural products. Unfortunately, clinical trials investigating OP treatment with natural products via epigenetic mechanisms are lacking. Given their potency and efficacy, natural products are promising candidates for preventing and treating OP, potentially reducing OP-related morbidity and mortality rates worldwide.
