# Supplementary material for: Therapeutic approach of natural products that treat osteoporosis by targeting epigenetic modulation
Source: Front Genet. 2023 May 23;14:1182363. doi: 10.3389/fgene.2023.1182363 (PMC10242146; doi:10.3389/fgene.2023.1182363)
Supplement: Supplementary file 1 [file Table1.docx]

**Supplementary Table 1. The outcomes and mechanisms of DNA/RNA methylation on OP from recent studies**

| **Types** | **Gene** | **Target / Pathway** | **Methyltransferase** | **Effects** | **References** |
| --- | --- | --- | --- | --- | --- |
| DNA methylation | BMP2 | Suppressed BMP2 expression | / | Inhibited bone formation | (Raje et al., 2019 ) |
|  | sFrp2 | Regulation of Wnt signaling pathway | / | Inhibited bone formation | (Peng et al., 2022) |
|  | JKAMP | Regulation of Wnt signaling pathway | / | Inhibited bone formation | (Peng et al., 2021 ) |
|  | MEPE,SOST, WIF1,DKK1 | Regulation of Wnt signaling pathway | / | Reduced BMD | (Reppe et al., 2017) |
|  | ZNF267, ABLIM2, RHOJ, CDKL5, PDCD1 | / | / | Reduced BMD | (Cheishvili et al., 2018) |
|  | SOST | Regulation the SP7, RUNX2, and ERa transactivation | / | Regulation of osteoblast proliferation and apoptosis | (Shan et al., 2019) |
|  | RANKL | Regulation RANKL and OPG transcription | [/](https://www-ncbi-nlm-nih-gov-443--bjmu.jitui.me/nuccore/NG_008990.1) | Regulation of osteoclastogenesis | (Delgado-Calle et al., 2012) |
|  | TBC1D8 | activated GTP enzyme | / | Regulation of osteoclastogenesis | (Ma QQ et al., 2018) |
|  | CDKN2A | Regulate the expression of ANRIL | / | Reduced BMC and BMD | (Curtis et al., 2017) |
|  | RXRA | / | / | Reduced offspring bone mass | (Curtis et al., 2019) |
|  | SOST | Regulation of Wnt signaling pathway | / | Inhibited bone formation | (Cao et al., 2019) |
|  | OPG, RANKL | Regulation the ratio of RANKL/OPG | / | Regulation osteoclast differentiation and maturation, enhance bone resorption and osteolysis | (Wang Y et al., 2020) |
|  | C/EBPα | Prevented PPARc binding | / | Regulation osteoclast differentiation | (Zhao QH et al., 2013) |
|  | KLF5 | Regulation of β-catenin expression | DNMT3B | Regulation osteogenic differentiation, inhibited bone formation | (Li LP et al., 2021) |
|  | FoxO3 | / | / | Regulation osteoblast aging and bone loss | (Lian et al., 2021a) |
|  | PPARγ2 | Regulation of PPARγ2 expression | / | Regulation osteoclast differentiation | (Zhang Y et al., 2015) |
|  | H19 | Inhibited Erk signaling pathway | / | Inhibited bone formation | (Li B et al., 2018) |
|  | IRF8 | / | / | Regulation of osteoclastogenesis | (Fang et al., 2016) |
|  | Shh | / | DNMT3B | Inhibit osteoblastogenesis of BMSCs | (Wang C et al.,2017) |
|  | SOST | Regulation of Wnt signaling pathway | DNMT3A | Inhibited bone formation | (Reppe et al., 2015) |
|  | C/EBPα | / | KDM7A | Promoted adipogenic differentiation , blocked osteogenic differentiation. | (Yang X et al., 2019) |
|  | H3K36me3 | / | SETD2 | Promoted adipogenic differentiation, blocked osteogeneic differentiation | (Wang L et al., 2018) |
|  | CD40 | / | / | Regulation of bone homeostasis, reduced BMD | (Panach et al., 2016) |
|  | sFRP-4 | Regulation of Wnt signaling pathway | / | Promoted osteoblastic inactivation | (Mori et al., 2014) |
| RNA methylation | m6A | / | Mettl3 | Promoted adipogenic differentiation, compromised osteogenic potential | (Wu et al., 2018) |
|  | m6A | / | FTO | Regulation osteogenic differentiation | (Wang J et al., 2021) |
|  | m6A | / | / | Regulation osteogenic differentiation | (Liu T et al., 2021) |
|  | m6A | / | METTL14 | Regulation osteogenic differentiation, regulation autophagy disturbance | (He et al., 2022) |
|  | m6A | / | FTO | Inhibited bone formation | (Chen LS et al., 2021) |
|  | m6A | / | METTL14 | Inhibited bone formation | (Wang F et al., 2022) |
|  | m6A | / | METTL14 | Inhibited bone formation | (Huang and Wang, 2022) |
|  | PPARγ | / | FTO | promoted the shift of osteoporotic BMSC fate to adipocyte and inhibited bone formation | (Shen et al., 2018) |
|  | ZIC1 | / | / | Reduced BMD | (Datta et al., 2022) |

“ / ” represents that there is no related content in the literature.

**Supplementary Table 2. The effects and mechanisms underlying the ncRNA-mediated regulation of OP**

| **Types** | **RNA** | **Target /pathway** | **Function** | **References** |
| --- | --- | --- | --- | --- |
| circRNA | circRNA 0001275 | Regulated miRNA-377/CDKN1B axis | Regulated osteoblast growth | (Xu Y et al., 2021) |
|  | circRNA 0001485 | Regulated targeting BMPR2 to activate the TGFβ-BMP pathway | promoted osteogenic differentiation | (Chen et al., 2022) |
|  | circRNA 0006766 | Regulated miRNA miRNA-4739 | Regulated osteogenic differentiation | (Guo Z et al., 2021) |
|  | circRNA 0062582 | Regulated miRNA-145/CBFB axis | Promoted osteogenic differentiation | (Li F et al., 2021) |
|  | circRNA 374b5p | Targeted regulationWnt3 AND Runx2 | Induced osteoblast differentiation | (Xu Y et al., 2020) |
|  | circRNA hsacirc 0006215 | Regulated miRNA-942-5p, RUNX2 and VEGF | Inhibited osteogenic differentiation | (Ji et al., 2021) |
|  | circRNA PVT1 | Regulated miRNA-30d-5p/ITGB3 axis | Regulated osteogenic differentiation of BMSCs | (Tan et al., 2022) |
|  | circRNA 19b | / | Regulated bone formation | (Sun M et al., 2019) |
|  | circRNA 0001795 | Regulated yes-associated protein 1 expression | Regulation osteogenic differentiation | (Li M et al., 2022) |
|  | circRNA 0006393 | Regulated miRNA‑145‑5p and FOXO1 | Inhibited bone formation | (Wang XB et al., 2019) |
|  | circRNA 0076906 | Regulated miRNA-1305/ OGN signaling pathway | Promoted osteogenic differentiation | (Wen et al., 2020) |
|  | circRNA 28313 | Regulated CircRNA_28313/miRNA-195a/CSF1 axis | Inhibited osteoblast differentiation | (Chen X et al., 2019) |
|  | circRNA circ 0000020 | Regulated circRNA_0000020/miRNA-142-5p/BMP2/SMAD-dependent signaling pathway | Regulation BMSCs osteogenic differentiation | (Zhou R et al., 2021) |
|  | circRNA 338 | Regulated of Runx2/Sox4/miRNA-338 positive feedback loop | Inhibited osteoblast differentiation | (Lin et al., 2019) |
| lncRNA | lnc RNA AK039312 and AK079370 | Regulated miRNA-199b-5p | Inhibited bone formation | (Yin et al., 2021) |
|  | lnc RNA colon | Regulated miRNA-34a-5p | Inhibited proliferation and differentiation of osteoblasts | (Hu et al., 2021) |
|  | lnc RNA HCG18 | Regulated miRNA-30a-5p/NOTCH1 axis | Inhibited BMSC osteogenic differentiation | (Che et al., 2020) |
|  | lnc RNA miRNAG | Regulated of miRNA-1897 | Induced osteoclastogenesis and bone resorption | (Ling L et al., 2019) |
|  | lnc RNA TRG AS1 | Regulated miRNA-802-mediated CAB39/AMPK/SIRT-1/NF-κB axis | Inhibited osteoblast differentiation | (Liu W et al., 2022) |
|  | lncRNA 00899 | Targeted regulationmiRNA-374a and RUNX2 expression | Promoted osteogenic differentiation | (Gao et al., 2021) |
|  | lncRNA 01119 | Regulated the Wnt pathway by targeting FZD4 | Inhibited osteogenic differentiation | (Gao et al., 2022) |
|  | lncRNA AK016739 | / | Inhibited osteoblast differentiation and bone formation | (Yin C et al., 2019) |
|  | lncRNA AK023948 | Regulated PI3K/AKT signaling pathway | Regulation the proliferation of osteoblasts | (Wang H et al., 2020) |
|  | lncRNA AK125437 | Regulated MAPK signaling pathway | Regulation the bone mineral density | (Wang H et al., 2020) |
|  | lncRNA BCAR4 | / | Promoted BMSC osteogenic differentiation | (Li DJ et al., 2020) |
|  | lncRNA BDNF AS | Regulated BDNF-AS signaling pathway | Inhibited osteoblast differentiation | (Feng et al., 2018) |
|  | lncRNA Bmncr | Regulated the RANML | Inhibited osteoclast differentiation | (Chen RS et al., 2019) |
|  | lncRNA Crnde | Regulated Wnt/β-catenin signaling pathway | Regulated bone formation | (Mulati et al., 2020) |
|  | lncRNA DANCR | Regulated Wnt/β-catenin signaling pathway | Suppressed osteogenic differentiation | (Wang CG et al., 2020) |
|  | lncRNA DANCR | Regulated miRNA-1301-3p/PROX1 axis | regulates osteogenic differentiation | (Weng et al., 2021) |
|  | lncRNA GAS5 | / | Increased fracture risk | (Visconti et al., 2020) |
|  | lncRNA GAS5 | DownRegulated miRNA-21 | Promote apoptosis of osteoclasts | (Cong et al., 2020) |
|  | lncRNA GAS5 | Via targeting miRNA-498 to regulate RUNX2 | Promoted osteogenic differentiation of MSCs | (Feng J et al., 2019) |
|  | lncRNA GAS5 | Regulated miRNA‑10a‑3p/VEGFA axis | Regulation angiogenesis | (Wu et al., 2021) |
|  | lncRNA HOTAIR | inhibition on miRNA-17-5p | Regulated fracture healing | (Su et al., 2021) |
|  | lncRNA HOTAIR | Regulated Wnt/β-catenin pathway | Inhibited osteogenic differentiation of BMSCs | (Shen et al., 2019) |
|  | lncRNA IGF2 AS | Upregulate KLK4 | Regulated osteogenic differentiation of BMSCs | (Tang et al., 2021) |
|  | lncRNA KCNQ1OT1 | Regulated of the miRNA-205-5p/RICTOR axis | Promoted osteogenic differentiation | (Yang JJ et al., 2022) |
|  | lncRNA KCNQ1OT1 | Regulated miRNA-214 | Inhibited Osteogenic Differentiation | (Wang CG et al., 2019) |
|  | lncRNA KCNQ1OT1 | Regulated miRNA-128-3p/NFAT5 axis | Inhibited osteoclast differentiation | (Zhang H et al., 2022) |
|  | lncRNA KCNQ1OT1 | Interacted miRNA-98-5p/Tbx5 axis | Promoted bone formation | (Wang FR et al., 2022) |
|  | lncRNA LINC00314 | Regulated the hsa-miRNA-129-5p/GRM5 axis via the Wnt signaling pathway | Regulated osteogenic differentiation | (Shi et al., 2020) |
|  | lncRNA LINC01535 | upregulated BMP2 expression | Promoted osteogenic differentiation | (Zhao Y et al., 2020) |
|  | lncRNA LINC02381 | Regulated KLF12-mediated Wnt4 transcriptional repression | Regulated osteogenic differentiation | (Zhao G et al., 2022) |
|  | lncRNA 000052 | Regulated the miRNA-96-5p-PIK3R1 axis | Regulation BMSC proliferation, migration, osteogenesis, and apoptosis | (Li M et al., 2020) |
|  | lncRNA LOXL1 AS1 | Regulated the miRNA-196a-5p/Hmga2 axis | Regulated osteogenic and adipocytic differentiation of BMSC | (Zhang L et al., 2020) |
|  | lncRNA MALAT1 | Regulated miRNA-34c/SATB2 axis | Enhanced osteoblast activity | (Yang X et al., 2019) |
|  | lncRNA MALAT1 | Regulated MAPK signaling pathway | Inhibited the osteogenic differentiation | (Zheng et al., 2019) |
|  | lncRNA MALAT1 | Targeted regulationmiRNA-143 | Regulated osteogenic differentiation | (Gao et al., 2018) |
|  | lncRNA MALAT1 | Regulated miRNA-217/AKT3 axis | Promoted osteogenic differentiation | (Song C et al., 2022) |
|  | lncRNA MEG3 | Targeted regulationmiRNA-133a-3p | Inhibited Osteogenic Differentiation of BMSCs | (Wang Q et al., 2017) |
|  | lncRNA MIAT | Regulated of AMPK/p38MAPK signaling pathway | Induced inflammatory cytokine secretion | (Li R et al., 2022) |
|  | lncRNA MIAT | Targeted regulationmiRNA-150-5p | Regulated the proliferation, apoptosis, and osteogenic differentiation | (Wang F et al., 2022) |
|  | lncRNA NEAT1 | Regulated LncRNA NEAT1/miRNA-29b-3p/BMP1 axis | Promoted osteoblast differentiation | (Zhang Y et al., 2019) |
|  | lncRNA ORlnc1 | Regulated miRNA-200b-3p/Foxo3 Pathway | promoted BMSCs pyroptosis | (Zhang L et al., 2021) |
|  | lncRNA PTCSC3 | / | Negatively regulates osteoblast apoptosis | (Liu X et al., 2022) |
|  | lncRNA RAD51 AS1 | Interaction with YBX1 | Promoted the proliferation and osteogenic differentiation | (Li et al., 2023) |
|  | lncRNA ROR | Regulated LncRNA ROR/miRNA-145-5p axis | Inhibited osteoblast proliferation | (Fu et al., 2021) |
|  | lncRNA RP11 84C13.1 | Regulated the miRNA-23b-3p/RUNX2 axis | Promoted osteogenic differentiation | (Yu H et al., 2021) |
|  | lncRNA SERPINB9P1 | Regulated miRNA-545-3p/SIRT6 signalling pathway | Accelerated osteogenic differentiation | (Wu et al., 2023) |
|  | lncRNA SNHG1 | Regulated of Opg | Enhanced adipogenic differentiation | (Yu X et al., 2022) |
|  | lncRNA SNHG1 | Modulated SFRP1/Wnt signaling pathway | Inhibited osteoblast differentiation and angiogenesis while promoting osteoclast formation | (Yu X et al., 2021) |
|  | lncRNA SNHG1 | Regulated p38 MAPK pathway | Attenuates Osteogenic Differentiation of BMSCs | (Jiang Y et al., 2019) |
|  | lncRNA SNHG14 | Regulated miRNA-185-5p/WISP2 axis | Regulated osteogenic differentiation | (Liu ZH et al., 2021) |
|  | lncRNA TERC | upregulate RUNX2 | Regulated bone formation | (Gao GC et al., 2020) |
|  | lncRNA TUG | Regulated miRNA-204/SIRT 1 signaling pathway | Regulated osteogenic differentiation of BMSCs | (Ouyang et al., 2022) |
|  | lncRNA UCA1 | Regulated BMP-2 expression | Regulated the proliferation and differentiation of osteoblasts | (Zhang RF et al., 2019) |
|  | lncRNA WT1 AS | Interactde with p53 | Regulated the apoptosis of osteoblasts | (Wang C et al., 2021) |
|  | lncRNA XIST | Regulated miRNA-590-3p/Tgif2 axis | Inhibited osteoblast differentiation | (Shao Y et al., 2021) |
|  | lncRNA XIST | Interacted with FUS | Inhibited osteoclast differentiation | (Zhang DW et al., 2022) |
|  | lncRNA XIST | / | Regulated osteogenic differentiation | (Chen X et al., 2019) |
|  | lncRNA XIXT | Targeted regulationmiRNA-30a-5p | Promoted Osteogenic Differentiation of BMSCs | (Zhang HL et al., 2019) |
|  | lncRNA ZBTB40 IT1 | Regulated miRNA-514a-3p/FOXO4 axis | Inhibited Osteogenic Differentiation | (Shi et al., 2022) |
|  | lncRNA ZBTB40 IT1 | / | Inhibited osteogenesis | (Mei et al., 2019) |
|  | lncRNA ZFAS1 | Upregulated miRNA-499-EPHA5 axis | Inhibited BMSC osteogenic differentiation | (Wu J et al., 2022) |
|  | lncRNA AK045490 | Via β-Catenin/TCF1/Runx2 Signaling Axis | Promoted osteoblast differentiation and bone formation | (Li D et al., 2019) |
|  | lncRNA AK137033 | Regulated of Wnt signaling pathway | Inhibited bone formation | (Peng et al., 2022) |
|  | lncRNA ANCR | Regulated the EZH2 and RUNX2 | Regulated bone formation | (Cai et al., 2019) |
|  | lncRNA ob1 | Up-regulated the expression of Osterix | Regulation osteoblast activity and bone formation | (Sun Y et al., 2019) |
|  | lncRNAp21 | Regulated Wnt/β-catenin signaling pathway | Enhanced osteogenic differentiation | (Yamg K et al., 2019) |
|  | lncRNA GAS5 | Regulated the miRNA-135a-5p/FOXO1 axis | Suppressed BMSC osteogenic differentiation | (Wang X et al., 2019) |
|  | lncRNA HOTAIR | Regulated miRNA miRNA-378g | Suppressed BMSC osteogenic differentiation | (Wang W et al., 2021) |
|  | lncRNA 22HG | Regulated PTEN/ AKT pathway | Promoted osteogenic differentiation | (Jin C et al., 2020) |
|  | lncRNA XIST | Regulated miRNA-29b-3p | Suppressed BMSC osteogenic differentiation | (Yu J et al., 2021) |
|  | lncRNA LOC100126784 and POM121L9P | Regulated miRNA-503-5p/SORBS1 Axis | Enhanced osteogenic differentiation | (Xu YY et al., 2021) |
| miRNA | miRNA 376c | Targeted regulationWnt3 and ARF-GEF-1 -facilitated augmentation of beta-catenin transactivation | Inhibited osteogenesis and Promoted bone loss | (Kureel et al., 2018) |
|  | miRNA Let 7a 5p | Regulated RNA KCNQ1OT1 and Participates | Inhibited osteoblast differentiation | (Alrashed et al., 2022) |
|  | miRNA 100 5p | Regulated fibroblast growth factor 21 | Inhibited osteoclastogenesis and bone resorption | (Zhou et al., 2019) |
|  | miRNA 103 3p t | Regulated miRNA-103-3p/METTL14/m6A signaling axis | Inhibited osteoblastic bone formation | (Sun et al., 2021) |
|  | miRNA 106b | Regulated targeting BMP2 | Inhibited osteoblastic differentiation and bone formation | (Liu et al., 2017) |
|  | miRNA 1224 5p | Regulated the Rap1 signaling target ADCY2 | Slowed osteoclast differentiation and promoted osteoblast differentiation | (Hu L C et al., 2022) |
|  | miRNA 124 | Via Targeting Rab27a | Attenuates Osteogenic Differentiation of BMSCs | (Tang L et al., 2017) |
|  | miRNA 124 3p | Regulated GSK-3β/β-catenin signaling pathway | Promoted BMSC osteogenesis | (Li Z et al., 2020) |
|  | miRNA 1249 5p | Targeted regulationPDX1 | Regulated osteogenic differentiation | (Yang XM et al., 2021) |
|  | miRNA 125a 5p | Targeted regulationTNFRSF1B | Promoted osteoclastogenesis | (Sun L et al., 2019) |
|  | miRNA 125b | / | Promoted cell proliferation and osteogenic differentiation of BMSCs | (Chen S et al., 2014) |
|  | miRNA 1263 | Regulated miRNA-1263/Mob1/Hippo signaling pathway | Inhibited BMSC apoptosis | (Yang BC et al., 2020) |
|  | miRNA 128 | Down-regulated SIRT6 expression | Inhibited the osteogenic differentiation | (Zhao J et al., 2019) |
|  | miRNA 128 3p | Regulated Wnt3a signaling | Regulated osteogenic differentiation | (Lin et al., 2021) |
|  | miRNA 1286 | Regulated FZD4 expression | Inhibited BMSC osteogenic differentiation | (Zhou JG et al., 2020) |
|  | miRNA 1297 | Targeted regulationWNT5A | Regulated osteogenic differentiation | (Wang Q et al., 2019) |
|  | miRNA 132 3p | / | Regulated osteogenic differentiation and osteogenesis | (Hu Z et al., 2020) |
|  | miRNA 133 | Regulated SLC39A1 expression | regulated osteogenic differentiation | (Lv et al., 2015) |
|  | miRNA 133a | / | Regulated Osteoclast Differentiation | (Li Z et al., 2018) |
|  | miRNA 133a | Regulated MAPK/ERK Signaling Pathway | Maintained the viability and balance between osteoblast and adipocyte differentiation of BMSC | (Wang G et al., 2021a) |
|  | miRNA 133a | Regulated MAPK/ERK signaling pathway | Regulated bone loss | (Wang G et al., 2021b) |
|  | miRNA 133a 5p | Regulated targeting the 3' UTR of RUNX2 | Inhibited osteoblast differentiation | (Zhang W et al., 2018) |
|  | miRNA 133b | Via Targeting GNB4 | Regulated Osteoclast Differentiation | (Wang J et al., 2021) |
|  | miRNA 134 5p | Regulated miRNA-134-5p/Itgb1/MAPK signaling pathway | Inhibited osteoclast differentiation | (Huang et al., 2022) |
|  | miRNA 135 5p | Targeted regulationHIF1AN | Promoted osteoblast differentiation | (Yin N et al., 2019) |
|  | miRNA 137 | Regulated RUNX2 | Regulated the bone mineral density | (Cai et al., 2020) |
|  | miRNA 138 5p | Regulated MACF1 | Inhibited osteoblast differentiation | (Chen Z et al., 2020) |
|  | miRNA 138 5p | Regulated MACF1 | Aggravated the decrease of aged osteoblast differentiation and led to worse bone loss | (Chen Z et al., 2020) |
|  | miRNA 139 3p | Targeted regulationELK1 and interacted with Lnc RNA ODSM | Regulated osteoblast differentiation and apoptosis | (Wang Y et al., 2018) |
|  | miRNA 139 5p | Regulated Wnt/Beta-Catenin Signaling Pathway | Promoted osteogenic differentiation of BMSCs | (Feng et al., 2020) |
|  | miRNA 1 3p | Targeted regulationsecreted frizzled-related protein 1 | promoted osteoclast activity and regulating bone resorption | (Gu H et al., 2020) |
|  | miRNA 140 3p | Downregulated MCF2L | Repressed preosteoblast viability and differentiation while promoted preosteoblast apoptosis | (Mao JH et al., 2019) |
|  | miRNA 141 | Regulated the Wnt/β-catenin pathway | Regulated the content of osteoprotegerin | (Liu TJ et al., 2020) |
|  | miRNA 142 | Regulated bone morphogenetic protein 2 | Regulated osteoblast differentiation | (Luo et al., 2020) |
|  | miRNA 143 | Regulated RANK, NF-κB and MAPK Signaling Pathways | Inhibited osteoclast formation | (He et al., 2020) |
|  | miRNA 143/145 | Regulated SOX2 transcription | Enhanced bone loss, inhibited retains bone | (Xu R et al., 2021) |
|  | miRNA 144 | DownRegulated expression of SFRP1 | Promoted the proliferation and differentiation | (Tang L et al., 2019) |
|  | miRNA 144 3p | Regulated the RANK | Regulated bone homeostasis | (Wang C et al., 2018) |
|  | miRNA 144 5p | Regulated miRNA-144-5p/IRS1/AKT axis | Regulated the migration, proliferation, and mineralization of osteoblasts | (Miao et al., 2022) |
|  | miRNA 146a | Suppressing RANKL/OPG and M-CSF | Attenuated bone loss | (Zhao J et al., 2019) |
|  | miRNA 146a | Regulated the Wnt/β‑catenin signaling pathway | Regulated BMD | (Liu et al., 2021b) |
|  | miRNA 148a | Regulated PI3K/AKT signaling by estrogen receptor α | Reduced apoptosis in osteoblasts | (Xiao et al., 2018) |
|  | miRNA 151a 3p | Regulated JAK2/STAT3 signaling pathway | Reduced bone density | (Fu Y et al., 2020) |
|  | miRNA 151a 3p | / | Regulated Osteoclast Differentiation | (He et al., 2021) |
|  | miRNA 151b | Down regulated Msx2 | Inhibited osteoblast differentiation | (Liu F et al., 2022) |
|  | miRNA 152 | Targeted regulationRICTOR | Regulated osteoblast differentiation | (Feng L et al., 2019) |
|  | miRNA 155 | Up-regulated LEPR | Suppressed repressed osteoclast activation and bone resorption of osteoclasts | (Mao Z et al., 2019) |
|  | miRNA 155 | Up-Regulated SIRT1 | Regulated osteoblastic differentiation | (Qu et al., 2020) |
|  | miRNA 15b | Regulated miRNA-15b/USP7/KDM6B | Inhibited osteoblast differentiation and autophagy | (Lu X et al., 2021) |
|  | miRNA 16 5p | Downregulated VEGFA expression | Inhibited bone formation | (Yu et al., 2020) |
|  | miRNA 17 3p | Downregulated Sox6 expression | Inhibit osteoblast differentiation | (Chen et al., 2020) |
|  | miRNA 181a | Regulated FasL protein expression | Regulated BMMSC‑induced CD4+T lymphocyte apoptosis | (Shao et al., 2018) |
|  | miRNA 182 | Regulated targeting Smad1 | Inhibited BMSC osteogenic differentiation | (Zheng et al., 2021) |
|  | miRNA 183 | Regulated Smad4 | Inhibited osteoblast differentiation | (Qin et al., 2021) |
|  | miRNA 185 | Regulated BMP/Smad Signaling Pathway | Promoted osteogenic differentiation and suppresses bone loss | (Cui et al., 2019) |
|  | miRNA 186 | / | Regulated bone formation | (Li L et al., 2021) |
|  | miRNA 186 | Regulated SIRT6 | Enhanced osteogenic differentiation | (Xiao J et al., 2020) |
|  | miRNA 187 | Targeted regulationBARX2 | accelerated osteoblastic differentiation | (Zhang J et al., 2021) |
|  | miRNA 187 3p | Suppressing cannabinoid receptor type 2 | Regulated osteoblast differentiation | (Xu et al., 2019) |
|  | miRNA 187 5p | Targeted regulationICAM1 | Regulated osteoblastic differentiation | (Sun et al., 2020) |
|  | miRNA 188 | / | Enhanced bone loss and fat accumulation in bone marrow | (Li et al., 2015) |
|  | miRNA 18a 3p | Regulated the glutamate AMPA receptor subunit 1 gene (GRIA1) | Promoted osteoporosis and contributes to fracture | (Zhao M et al., 2022) |
|  | miRNA 1906 | Down-regulated the TLR4/MyD88/NF-κB pathway | Attenuated bone loss | (Xie et al., 2021) |
|  | miRNA 195 5p | Activation BMP-2/SMAD/Akt/RUNX2 pathway via targeting SMURF1 | Enhanced osteogenic differentiation of osteoclast | (Ye et al., 2021) |
|  | miRNA 196a | Regulated GNAS-dependent Hedgehog signaling pathway | accelerates osteogenic differentiation | (Zhong et al., 2019) |
|  | miRNA 197 3p | Down-Regulated KLF 10 | Promoted osteoblast differentiation | (You et al., 2021) |
|  | miRNA 199a 3p | Via targeting Kdm3a | Enhanced osteogenic differentiation | (Wu JC et al., 2021) |
|  | miRNA 199a 5p | Targeted regulationTET2 | Promoted osteoblast differentiation | (Qi XB et al., 2019) |
|  | miRNA 19a 3p | Targeted regulationHDAC4 | Promoted the osteogenic differentiation of hMSCs | (Chen R et al., 2019) |
|  | miRNA 19b 3p | / | Aggravated bone loss and suppressed BMSC-derived osteoblast differentiation | (Liu D et al., 2022) |
|  | miRNA 19b 3p | Interacting with LncRNA H19 | Promoted cell proliferation and osteogenic differentiation of BMSCs | (Gao X et al., 2020) |
|  | miRNA 19b 3p | Regulated IGF-1 expression | Regulated osteoblast differentiation | (Yu Y et al., 2022) |
|  | miRNA 200a 3p | Targeted regulationglutaminase | Inhibited osteogenic differentiation of BMSCs | (Lv et al., 2019) |
|  | miRNA 203 | Regulated DKK1 expression | Promoted osteogenic differentiation | (Xia et al., 2018) |
|  | miRNA 203 | DownRegulated DKK1 expression | Regulated of from osteogenic differentiation to adipogenic differentiation | (Qiao et al., 2018) |
|  | miRNA 205 5p | Regulated RUNX2 | Inhibited osteoblast differentiation | (Huang M et al., 2020) |
|  | miRNA 206 | Regulated of HDAC4 | Regulation osteoblast cell proliferation and apoptosis | (Lu Z et al., 2021) |
|  | miRNA 21 | Targeted regulationPTEN | Regulated osteogenic and adipogenic differentiation | (Zhou Y et al., 2021) |
|  | miRNA 21 | Regulated BMP9/Smad signaling pathway | Regulated osteoblastic differentiation | (Song et al., 2015) |
|  | miRNA 21 | / | Induced proliferation and differentiation of MSCs | (Hu LS et al., 2022) |
|  | miRNA 210 | Regulated VEGF expression | Promoted osteoblast differentiation | (Liu et al., 2015) |
|  | miRNA 211 5p | Regulated dual specific phosphatase 6 | Promoted osteogenic differentiation | (Wang H et al., 2022) |
|  | miRNA 212 and miRNA 384 | Targeted regulationRunx2 | Promoted osteogenic differentiation | (Zhang Y et al., 2020) |
|  | miRNA 214 | Regulated targets ATF4 | Inhibited bone formation | (Wang et al., 2013) |
|  | miRNA 214 | Via Targeting FGFR1 | Attenuates Osteogenic Differentiation of BMSCs | (Yang et al., 2016) |
|  | miRNA 214 | Regulated Pten/PI3k/Akt pathway | promoted osteoclastogenesis | (Zhao et al., 2015) |
|  | miRNA 214 | Suppressed β-catenin expression and attenuated Wnt/β-catenin signaling pathway | Inhibited osteogenic differentiation of BMSCs | (Li et al., 2017) |
|  | miRNA 214 | Targeted regulationATF4 | against apoptosis and suppressed oxidative stress | (Lu et al., 2017) |
|  | miRNA 214 3p | Regulated BMP/Smad signaling pathway | Delays fracture healing | (Zhou LG et al., 2019) |
|  | miRNA 214 5p | Regulated miRNA-214-5p/TGF-β/Smad2 signaling pathway | alters adipogenic differentiation | (Qiu et al., 2018) |
|  | miRNA 215 5p | Regulated the XIAP | Regulated osteogenic differentiation | (Yin Z et al., 2022) |
|  | miRNA 216a | Regulated c-Cbl-mediated PI3K/AKT pathway | Promoted osteoblast differentiation and enhances bone formation | (Li H et al., 2015) |
|  | miRNA 218 | Repressed the Nuclear Factor-κB Signaling Pathway and Targeting Tumor Necrosis Factor Receptor 1 | Regulated Osteoclastogenic Differentiation | (Wang W et al., 2018) |
|  | miRNA 218 | Regulated Wnt/β-catenin signaling pathway | Promoted osteogenic differentiation | (Karimi et al., 2020) |
|  | miRNA 218 | Regulated p38MAPK-c-Fos-NFATc1 signaling pathway | Regulated for osteoclastogenesis and bone resorption | (Qu et al., 2015) |
|  | miRNA 218 and miRNA 618 | / | Inhibited osteoclast differentiation | (Wang WW et al., 2017) |
|  | miRNA 218 5p | / | Promoted the osteoblast differentiation | (Kou et al., 2020) |
|  | miRNA 221 | Regulated RUNX2 | Regulated osteoblast differentiation | (Zhang Y et al., 2017) |
|  | miRNA 221 5p | Regulated miRNA-221-5p/Smad3 axis | Suppressed osteoclastogenesis | (Guo et al., 2022) |
|  | miRNA 23a | Regulated of the OB-OC-MΦ axis | Inhibited osteogenesis, promoting bone resorption and inflammatory polarization of macrophages | (Ma TL et al., 2022) |
|  | miRNA 23b | Regulated RUNX2 | Enhanced osteogenic differentiation | (Deng et al., 2018) |
|  | miRNA 25 3p | Regulated nuclear factor I X | Reduced bone formation | (Huang Y et al., 2020) |
|  | miRNA 26a | Regulatedthe expression of serum IGF-1 | suppress the proliferation of chondrocytes and promote their apoptosis | (Yuan et al., 2021) |
|  | miRNA 26b | Regulated GSK3β/β-catenin pathway | promoted BMSC osteogenesis | (Hu et al., 2019) |
|  | miRNA 27a | Targeted regulationMef2c | Regulated of MSCs from osteogenic differentiation to adipogenic differentiation | (You et al., 2014) |
|  | miRNA 27a 3p | / | Promoted osteogenic differentiation | (Fu et al., 2019) |
|  | miRNA 27a 3p | Regulated CRY2/ERK1/2 axis | Enhanced osteogenic differentiation | (Ren et al., 2021) |
|  | miRNA 27a 3p | Targeted regulationosterix | Regulated osteogenic differentiation | (Xu Y et al., 2020) |
|  | miRNA 291a 3p | Via targeting DKK1 | Promoted osteogenic differentiation of BMSCs | (Li ZH et al., 2020) |
|  | miRNA 29a | Regulated of FoxO3 | Regulation osteoblast aging and bone loss | (Lian et al., 2021a) |
|  | miRNA 29a | Regulated PCAF-mediated RANKL and CXCL12 | Inhibited osteoclast formation | (Lian et al., 2019) |
|  | miRNA 29a | Targeted regulationLeptin | Regulated brown/beige adipocyte formation of osteogenic progenitor cells to preserve bone anabolism | (Lian et al., 2021b) |
|  | miRNA 29a | Regulated TNFSF13b | Enhanced bone loss | (Wu et al., 2019) |
|  | miRNA 29c 3p | Regulated Dvl2 expression | Reduced bone loss | (Cao et al., 2021) |
|  | miRNA 301 b | Regulated CYDR/NF-κB signaling pathway | Induced bone loss | (Zhu et al., 2020) |
|  | miRNA 30a 3p | Regulated the SFRP1 | Promoted bone formation | (Liu HP et al., 2019) |
|  | miRNA 320a | Regulated Wnt/β-catenin signaling pathway | Suppressed osteogenic differentiation | (Wang CG et al., 2020) |
|  | miRNA 320a | Reduced MAP9 and inhibited PI3K/AKT signaling pathway | Enhanced osteogenic differentiation and promoted MC3T3-E1 cells apoptosis | (Kong et al., 2019) |
|  | miRNA 330 5p | Activation Bgn-mediated BMP/Smad pathway | Inhibited bone loss | (Jin SL et al., 2020) |
|  | miRNA 335 5p | / | Inhibited osteoblast apoptosis | (Li et al., 2016) |
|  | miRNA 337 | Regulatedn of Rap1A | Regulated osteogenic differentiation | (Liu S et al., 2021) |
|  | miRNA 338 3p | Targeted regulationRunx2 and Fgfr2 | Regulated osteogenic differentiation | (Liu et al., 2014) |
|  | miRNA 338 3p | Regulated RANKL targeting | Inhibit osteoclast formation | (Zhang XH et al., 2016) |
|  | miRNA 338 3p | Targeted regulationPCSK5 | Inhibited osteoblastic differentiation and osteogenesis | (Tong et al., 2021) |
|  | miRNA 339 | Targeted regulationDLX5 | Promoted osteogenic differentiation of BMSCs | (Zhou J et al., 2019) |
|  | miRNA 340 5p | Regulated of runt-related transcription factor-2 (RUNX2) | Promoted osteogenic differentiation | (Wang X et al., 2021) |
|  | miRNA 342 3p | Regulated of ATF3 | Regulation osteogenic differentiation | (Han et al., 2018) |
|  | miRNA 34c | Targeted regulationLGR4 | Promoted osteoclast differentiation | (Cong et al., 2017) |
|  | miRNA 363 3p | Regulated PTEN/PI3K/AKT signaling pathway | Promoted osteoclastogenesis and Inhibited osteogenic differentiation | (Li M et al., 2019) |
|  | miRNA 365 | Regulated Targeting HDAC4 | Ameliorated DEX-induced suppression of cell viability and osteogenesis | (Xu et al., 2017) |
|  | miRNA 3653 3p | Modulated circRNA_0003865 | Inhibited osteoblast differentiation | (Wang X et al., 2021) |
|  | miRNA 365a 3p | Regulated targeting RUNX2 | Inhibited osteogenic differentiation | (Cheng et al., 2019) |
|  | miRNA 373 | / | Regulated osteogenic differentiation | (Li LY et al., 2019) |
|  | miRNA 375 3p | Regulated LRP5 and β-catenin | Regulated bone formation | (Sun et al., 2017) |
|  | miRNA 378 | Targeted regulationCASP3 and activated PI3K/Akt signaling pathway | Regulated osteoblastic differentiation | (You et al., 2016) |
|  | miRNA 384 5p | Regulated targeting Gli2 | Enhanced bone loss | (Li X et al., 2019) |
|  | miRNA 431 5p | / | Suppressed osteoblastic differentiation and promoted adipogenic differentiation | (Zhi et al., 2021) |
|  | miRNA 433 3p | Regulated DKK1 expression | Induced osteoblasts differentiation. | (Tang X et al., 2017) |
|  | miRNA 449b 5p | Targeted regulationSatb2 | Inhibit osteogenic differentiation | (Li JY et al., 2019) |
|  | miRNA 450b | Regulated targeting BMP3 | Regulated osteogenic differentiation | (Fan et al., 2018) |
|  | miRNA 451a | Regulated Bmp6 signaling pathway | Suppressed osteogenic differentiation and enhanced bone loss | (Lu et al., 2019) |
|  | miRNA 455 3p | Regulated the HDAC2 | Promoted osteogenic differentiation | (Ma et al., 2022) |
|  | miRNA 455 3p | Regulated Nrf2/ARE signaling pathway | Suppressed oxidative stress and promoting osteoblasts growth | (Zhang S et al., 2018) |
|  | miRNA 4739 | Regulated miRNA-4739/DLX3 Axis | Regulated osteogenic differentiation of BMSCs | (Li D et al., 2021) |
|  | miRNA 483 3p | Regulated Dikkopf 2 (DKK2) and the Wnt signaling pathway | Promoted the osteogenesis | (Zhou B et al., 2020) |
|  | miRNA 483 5p | / | Promoted osteoclast differentiation | (Li K et al., 2020) |
|  | miRNA 483 5p | Regulated targeting SATB2 and PI3K/AKT pathway | Inhibited osteoblast differentiation, Reduced BMD | (Zhao F et al., 2021) |
|  | miRNA 485 5p | Regulated the Osterix | Promoted osteogenic differentiation | (Zhang SY et al., 2018) |
|  | miRNA 486 3p | Regulated CTNNBIP1 and activated the Wnt/β-catenin signaling pathway | Regulated osteogenic differentiation | (Zhang Z et al., 2021) |
|  | miRNA 491 3p | Targeted regulationCTSS | Increased bone cell viability and differentiation and inhibited their apoptosis | (Hu WX et al., 2020) |
|  | miRNA 497 | Regulated TGF-β1/Smads signalling pathway | Regulated osteoblast proliferation | (Gu Z et al., 2020) |
|  | miRNA 497 5p | Regulated HMGA2-mediated JNK signaling pathway | Regulated osteoblast differentiation | (Zhao H et al., 2020) |
|  | miRNA 503 | Regulated the RANK | Inhibited osteoclastogenesis and bone resorption | (Chen C et al., 2014) |
|  | miRNA 505 | Regulated RUNX2 | Regulated osteogenic differentiation | (Li W et al., 2020) |
|  | miRNA 506 3p | Regulated RANKL/NFATc1 signaling pathway | Alleviated uncontrolled osteoclastogenesis | (Dinesh et al., 2020) |
|  | miRNA 542 3p | Regulated the SFRP1 | Induced osteoblast differentiation | (Zhang X et al., 2018) |
|  | miRNA 548 3p | Targeted regulationSTAT1 and MAFB | Inhibited proliferation, migration and invasion | (Ramírez-Salazar et al., 2020) |
|  | miRNA 577 | Regulated the TSHR | Aggravates bone loss and bone remodeling | (Xu et al., 2022) |
|  | miRNA 579 3p | Regulated Sirt1 | regulated osteogenic differentiation | (Luo et al., 2019) |
|  | miRNA 582 3p | / | Regulation osteoclast differentiation | (Yin J et al., 2022) |
|  | miRNA 655 3p | Targeted regulationLSD1 and activated BMP-2/Smad signaling pathway | Enhanced osteogenic differentiation | (Wang XJ et al., 2020) |
|  | miRNA 664 3p | Targeted regulationSmad4 and Osterix | suppresses osteoblast differentiation and impairs bone formation | (Xu Y et al., 2021a) |
|  | miRNA 664a 5p | Down-Regulated HMGA2 | Regulated osteogenic differentiation of BMSCs | (Zhang Y et al., 2020) |
|  | miRNA 765 | Regulated BMP6/Smad1/5/9 signaling pathway | Inhibited osteogenic differentiation | (Wang T et al., 2020) |
|  | miRNA 874 | Regulated Hedgehog signaling pathway | Promoted the proliferation and differentiation of osteoblasts | (Lin et al., 2018) |
|  | miRNA 920 | Regulated targeting HOXA7 | Inhibited Osteogenic Differentiation | (Zha et al., 2020) |
|  | miRNA 92b 5p | Targeted regulationICAM-1 | Regulated proliferation and differentiation of BMSCs | (Li Y et al., 2019) |
|  | miRNA 935 | / | Enhanced osteoblast proliferation and differentiation | (Zhang Y et al., 2021) |
|  | miRNA 9 5p | Regulated the Wnt3a | Inhibited osteogenesis and promoted adipogenesis | (Zhang HG et al., 2019) |
|  | miRNA 96 | / | Regulation BMSCs osteogenic differentiation | (Liu et al., 2018) |
|  | miRNA 98 5p | Regulated PI3K/AKT/GSK3β signaling pathway | Suppressed of preosteoblast viability and differentiation | (Zheng et al., 2022) |
|  | miRNA 99b 5p | Targeted regulationFGFR3 | Inhibited bone formation | (Ding et al., 2021) |
| piRNA | piRNA 36741 | Regulated METTL3-mediated m6A methylation of BMP2 transcripts | Regulation osteogenic differentiation | (Liu J et al., 2021) |
|  | PiRNA 63049 | Regulated Wnt/β-catenin signaling pathway | Inhibited bone formation | (Chen G et al., 2021) |

“ / ” represents that there is no related content in the literature.

**Supplementary Table 3. The outcomes and mechanisms of histone modifications on OP**

| **Types** | **Histone name** | **Target** | **Function** | **References** |
| --- | --- | --- | --- | --- |
| acetylation | H3K14ac | NAP1L2, SIRT3 | Regulated bone formation | (Hu M et al., 2022) |
|  | H3K9ac and H3K27ac | ACLY | Regulated osteoclast differentiation | (Guo Q et al., 2021) |
|  | H3K4ac, H3K14ac and H3K18ac | ZEB1 | Promoted angiogenesis-dependent bone formation | (Fu R et al., 2020) |
|  | H3ac | / | Regulated osteoblast differentiation | (Abuna et al., 2021) |
|  | H3K9ac | Wnt | Inibited osteogenic differentiation | (Jing et al., 2018) |
|  | H3K9/K14ac and H4K12ac | Runx3 and Osx | Regulated bone formation | (Zhang YX et al., 2015) |
|  | H27K1ac | GC-IGF1 | Mediated osteogenic dysdifferentiation and adult osteoporosis susceptibility | (Shangguan et al., 2022) |
|  | H3K9ac | / | Regulated osteogenic differentiation | (Zhang P et al., 2016) |
|  | H4 | / | Regulated inflammation | (Pucci et al., 2019) |
|  | H3K9/K14ac and H4K12ac | Runx2 | Regulated bone loss | (Ma et al., 2021) |
|  | H3 | Runx2 | Regulated osteoblast differentiation and bone formation | (Schroeder and Westendorf, 2005) |
|  | H4 | Akt | Regulated osteoblast differentiation | (Dudakovic et al., 2013) |
| methylation | H3K27me | Runx4 and Osx | Regulated bone formation | (Zhang YX et al., 2015) |
|  | H3K9 and H3K27 | α-KG | Regulated bone formation | (Wang Y et al., 2020) |
|  | H79K264me | / | Regulated osteoclast differentiation | (Gao et al., 2018) |
|  | H3K9me | C/EBPβ and Wnt | Regulated adipogenic and osteogenic differentiation | (Qi Q et al., 2019) |
|  | H3K36me2 and H3K27me3 | NSD2 | Regulated osteogenic differentiation | (Xie et al., 2022) |
|  | H3K27me3 and H3K9me3 | HOX and DLX | Regulated osteogenic differentiation | (Ye et al., 2012) |
|  | H3K27me3 | P16 | Regulated osteoblastic bone formation, osteocyte senescence, SASP, and osteoclastic bone resorption | (Yang R et al., 2020) |
|  | H3K4me1/2 | WNT7B and BMP2 | Regulated bone mass | (Sun et al., 2018) |
| phosphorylation | H2AX | FABP3 | Regulated bone-fat balance | (Liu ZZ et al., 2021) |
| ubiquitylation | H2Bub1 and H3K4me3 | RANKL | Regulated osteoblast differentiation | (Najafova et al., 2021) |

“ / ” represents that there is no related content in the literature.

# References

Abuna, R. P. F., Almeida, L. O., Souza, A. T. P., Fernandes, R. R., Sverzut, T. F. V., Rosa, A. L., et al. (2021). Osteoporosis and osteoblasts cocultured with adipocytes inhibit osteoblast differentiation by downregulating histone acetylation. *J. Cell. Physiol.* 236 (5), 3906-3917. doi:10.1002/jcp.30131

Alrashed, M. M., Alshehry, A. S., Ahmad, M., He, J., Wang, Y., and Xu, Y. (2022). miRNA Let-7a-5p targets RNA KCNQ1OT1 and participates in osteoblast differentiation to improve the development of osteoporosis. *Biochem. Genet.* 60 (1), 370-381. doi:10.1007/s10528-021-10105-3

Cai, N., Li, C., and Wang, F. (2019). Silencing of LncRNA-ANCR promotes the osteogenesis of osteoblast cells in postmenopausal osteoporosis via targeting EZH2 and RUNX2. *Yonsei Med. J.* 60 (8), 751-759. doi:10.3349/ymj.2019.60.8.751

Cai, W. L., Zeng, W., Zhu, B. Y., Liu, H. H., and Liu, J. L. (2020). MiR-137 affects bone mineral density in osteoporosis rats through regulating RUNX2. *Eur. Rev. Med. Pharmacol. Sci.* 24 (3), 1023-1029. doi:10.26355/eurrev_202002_20152

Cao, Y., Qiu, Y., Liu, M. X., Hu, Y., and Chen, F. W. (2021). MiR-29c-3p reduces bone loss in rats with diabetic osteoporosis via targeted regulation of Dvl2 expression. *Eur. Rev. Med. Pharmacol. Sci.* 25 (2), 636-642. doi:10.26355/eurrev_202101_24625

Cao, Y., Wang, B., Wang, D., Zhan, D., Mai, C., Wang, P., et al. (2019). Expression of sclerostin in osteoporotic fracture patients is associated with DNA methylation in the CpG island of the SOST gene. *Int. J. Genomics* 2019, 7076513. doi:10.1155/2019/7076513

Che, M., Gong, W., Zhao, Y., and Liu, M. (2020). Long noncoding RNA HCG18 inhibits the differentiation of human bone marrow-derived mesenchymal stem cells in osteoporosis by targeting miR-30a-5p/NOTCH1 axis. *Mol. Med.* 26 (1), 106. doi:10.1186/s10020-020-00219-6

Cheishvili, D., Parashar, S., Mahmood, N., Arakelian, A., Kremer, R., Goltzman, D., et al. (2018). Identification of an epigenetic signature of osteoporosis in blood DNA of postmenopausal women. *J. Bone Miner. Res.* 33 (11), 1980-1989. doi:10.1002/jbmr.3527

Chen, C., Cheng, P., Xie, H., Zhou, H. D., Wu, X. P., Liao, E. Y., et al. (2014). MiR-503 regulates osteoclastogenesis via targeting RANK. *J. Bone Miner. Res.* 29 (2), 338-347. doi:10.1002/jbmr.2032

Chen, G., Wang, S., Long, C., Wang, Z., Chen, X., Tang, W., et al. (2021). PiRNA-63049 inhibits bone formation through Wnt/β-catenin signaling pathway. *Int. J. Biol. Sci.* 17 (15), 4409-4425. doi:10.7150/ijbs.64533

Chen, L. S., Zhang, M., Chen, P., Xiong, X. F., Liu, P. Q., Wang, H. B., et al. (2021). The m^6^A demethylase FTO promotes the osteogenesis of mesenchymal stem cells by downregulating PPARG. *Acta Pharmacol. Sin.* 43 (5), 1311-1323. doi:10.1038/s41401-021-00756-8

Chen, N., Wu, D., Li, H., Liu, Y., and Yang, H. (2020). MiR-17-3p inhibits osteoblast differentiation by downregulating Sox6 expression. *FEBS Open Bio* 10 (11), 2499-2506. doi:10.1002/2211-5463.12979

Chen, R. S., Zhang, X. B., Zhu, X. T., and Wang, C. S. (2019). LncRNA Bmncr alleviates the progression of osteoporosis by inhibiting RANML-induced osteoclast differentiation. *Eur. Rev. Med. Pharmacol. Sci.* 23 (21), 9199-9206. doi:10.26355/eurrev_201911_19411

Chen, R., Qiu, H., Tong, Y., Liao, F., Hu, X., Qiu, Y., et al. (2019). MiRNA-19a-3p alleviates the progression of osteoporosis by targeting HDAC4 to promote the osteogenic differentiation of hMSCs. *Biochem. Biophys. Res. Commun.* 516 (3), 666-672. doi:10.1016/j.bbrc.2019.06.083

Chen, S. C., Jiang, T., Liu, Q. Y., Liu, Z. T., Su, Y. F., and Su, H. T. (2022). Hsa_circ_0001485 promoted osteogenic differentiation by targeting BMPR2 to activate the TGF β-BMP pathway. *Stem Cell Res. Ther.* 13 (1), 453. doi:10.1186/s13287-022-03150-1

Chen, S., Yang, L., Jie, Q., Lin, Y. S., Meng, G. L., Fan, J. Z., et al. (2014). MicroRNA‑125b suppresses the proliferation and osteogenic differentiation of human bone marrow‑derived mesenchymal stem cells. *Mol. Med. Rep.* 9 (5), 1820-1826. doi:10.3892/mmr.2014.2024

Chen, X., Ouyang, Z., Shen, Y., Liu, B., Zhang, Q., Wan, L., et al. (2019). CircRNA_28313/miR-195a/CSF1 axis modulates osteoclast differentiation to affect OVX-induced bone absorption in mice. *RNA Biol.* 16 (9), 1249-1262. doi:10.1080/15476286.2019.1624470

Chen, X., Yang, L., Ge, D., Wang, W., Yin, Z., Yan, J., et al. (2019). Long non-coding RNA XIST promotes osteoporosis through inhibiting bone marrow mesenchymal stem cell differentiation. *Exp. Ther. Med.* 17 (1), 803-811. doi:10.3892/etm.2018.7033

Chen, Z., Zhao, F., Liang, C., Hu, L., Li, D., Zhang, Y., et al. (2020). Silencing of miR-138-5p sensitizes bone anabolic action to mechanical stimuli. *Theranostics* 10 (26), 12263-12278. doi:10.7150/thno.53009

Cheng, F., Yang, M. M., and Yang, R. H. (2019). MiRNA-365a-3p promotes the progression of osteoporosis by inhibiting osteogenic differentiation via targeting RUNX2. *Eur. Rev. Med. Pharmacol. Sci.* 23 (18),7766-7774. doi: 10.26355/eurrev_201909_18986

Cong, C., Tian, J., Gao, T., Zhou, C., Wang, Y., Cui, X., et al. (2020). lncRNA GAS5 is upregulated in osteoporosis and downregulates miR-21 to promote apoptosis of osteoclasts. *Clin. Interv. Aging* 15, 1163-1169. doi:10.2147/CIA.S235197

Cong, F., Wu, N., Tian, X., Fan, J., Liu, J., Song, T., et al. (2017). MicroRNA-34c promotes osteoclast differentiation through targeting LGR4. *Gene* 610, 1-8. doi:10.1016/j.gene.2017.01.028

Cui, Q., Xing, J., Yu, M., Wang, Y., Xu, J., Gu, Y., et al. (2019). Mmu-miR-185 depletion promotes osteogenic differentiation and suppresses bone loss in osteoporosis through the Bgn-mediated BMP/Smad pathway. *Cell Death Dis*. 10 (3), 172. doi:10.1038/s41419-019-1428-1

Curtis, E. M., Krstic, N., Cook, E., D'Angelo, S., Crozier, S. R., Moon, R. J., et al. (2019). Gestational vitamin D supplementation leads to reduced perinatal RXRA DNA methylation: Results from the MAVIDOS trial. *J. Bone Miner. Res.* 34 (2), 231-240. doi:10.1002/jbmr.3603

Curtis, E. M., Murray, R., Titcombe, P., Cook, E., Clarke-Harris, R., Costello, P., et al. (2017). Perinatal DNA methylation at CDKN2A is associated with offspring bone mass: Findings from the southampton women's survey. *J. Bone Miner. Res.* 32 (10), 2030-2040. doi:10.1002/jbmr.3153

Datta, H. K., Kringen, M. K., Tuck, S. P., Salpingidou, G., Olstad, O. K., Gautvik, K. M., et al. (2022). Mechanical-stress-related epigenetic regulation of ZIC1 transcription factor in the etiology of postmenopausal osteoporosis. *Int. J. Mol. Sci.* 23 (6), 2957. doi:10.3390/ijms23062957

Delgado-Calle, J., Sañudo, C., Fernández, A. F., García-Renedo, R., Fraga, M. F., and Riancho, J. A. (2012). Role of DNA methylation in the regulation of the RANKL-OPG system in human bone. *Epigenetics* 7 (1), 83-91. doi:10.4161/epi.7.1.18753

Deng, L., Hu, G., Jin, L., Wang, C., and Niu, H. (2018). Involvement of microRNA-23b in TNF-α-reduced BMSC osteogenic differentiation via targeting runx2. *J. Bone Miner. Metab.* 36 (6), 648-660. doi:10.1007/s00774-017-0886-8

Dinesh, P., Kalaiselvan, S., Sujitha, S., and Rasool, M. (2020). miR-506-3p alleviates uncontrolled osteoclastogenesis via repression of RANKL/NFATc1 signaling pathway. *J. Cell. Physiol.* 235 (12), 9497-9509. doi:10.1002/jcp.29757

Ding, M., Liu, B., Chen, X., Ouyang, Z., Peng, D., and Zhou, Y. (2021). MiR-99b-5p suppressed proliferation of human osteoblasts by targeting FGFR3 in osteoporosis. *Hum. Cell* 34 (5), 1398-1409. doi:10.1007/s13577-021-00567-3

Dudakovic, A., Evans, J. M., Li, Y., Middha, S., McGee-Lawrence, M. E., van Wijnen, A. J., et al. (2013). Histone deacetylase inhibition promotes osteoblast maturation by altering the histone H4 epigenome and reduces AKT phosphorylation. *J. Biol. Chem.* 288 (40), 28783-28791. doi:10.1074/jbc.M113.489732

Fan, L., Fan, J., Liu, Y., Li, T., Xu, H., Yang, Y., et al. (2018). miR-450b promotes osteogenic differentiation in vitro and enhances bone formation in vivo by targeting BMP3. *Stem Cells Dev.* 27 (9), 600-611. doi:10.1089/scd.2017.0276

Fang, C., Qiao, Y., Mun, S. H., Lee, M. J., Murata, K., Bae, S., et al. (2016). Cutting edge: EZH2 promotes osteoclastogenesis by epigenetic silencing of the negative regulator IRF8. *J. Immunol.* 196 (11), 4452-4456. doi:10.4049/jimmunol.1501466

Feng, J., Wang, J. X., and Li, C. H. (2019). LncRNA GAS5 overexpression alleviates the development of osteoporosis through promoting osteogenic differentiation of MSCs via targeting microRNA-498 to regulate RUNX2. *Eur. Rev. Med. Pharmacol. Sci.* 23 (18), 7757-7765. doi:10.26355/eurrev_201909_18985

Feng, L., Xia, B., Tian, B. F., and Lu, G. B. (2019). MiR-152 influences osteoporosis through regulation of osteoblast differentiation by targeting RICTOR. *Pharm. Biol.* 57 (1), 586-594. doi:10.1080/13880209.2019.1657153

Feng, X., Lin, T., Liu, X., Yang, C., Yang, S., and Fu, D. (2018). Long non-coding RNA BDNF-AS modulates osteogenic differentiation of bone marrow-derived mesenchymal stem cells. *Mol. Cell. Biochem.* 445 (1-2), 59-65. doi:10.1007/s11010-017-3251-2

Feng, Y., Wan, P., Yin, L., and Lou, X. (2020). The Inhibition of MicroRNA-139-5p promoted osteoporosis of bone marrow-derived mesenchymal stem cells by targeting Wnt/Beta-catenin signaling pathway by NOTCH1. *J. Microbiol. Biotechnol.* 30 (3), 448-458. doi:10.4014/jmb.1908.08036

Fu, R., Lv, W. C., Xu, Y., Gong, M. Y., Chen, X. J., Jiang, N., et al. (2020). Endothelial ZEB1 promotes angiogenesis-dependent bone formation and reverses osteoporosis. *Nat. Commun.* 11 (1), 460. doi:10.1038/s41467-019-14076-3

Fu, Y. C., Zhao, S. R., Zhu, B. H., Guo, S. S., and Wang, X. X. (2019). MiRNA-27a-3p promotes osteogenic differentiation of human mesenchymal stem cells through targeting ATF3. *Eur. Rev. Med. Pharmacol. Sci.* 23 (3 Suppl), 73-80. doi:10.26355/eurrev_201908_18632

Fu, Y., Hu, X., Gao, Y., Li, K., Fu, Q., Liu, Q., et al. (2021). LncRNA ROR/miR-145-5p axis modulates the osteoblasts proliferation and apoptosis in osteoporosis. *Bioengineered* 12 (1), 7714-7723. doi:10.1080/21655979.2021.1982323

Fu, Y., Xu, Y., Chen, S., Ouyang, Y., and Sun, G. (2020). MiR-151a-3p promotes postmenopausal osteoporosis by targeting SOCS5 and activating JAK2/STAT3 signaling. *Rejuvenation Res*. 23 (4), 313-323. doi:10.1089/rej.2019.2239

Gao, G. C., Yang, D. W., and Liu, W. (2020). LncRNA TERC alleviates the progression of osteoporosis by absorbing miRNA-217 to upregulate RUNX2. *Eur. Rev. Med. Pharmacol. Sci.* 24 (2), 526-534. doi:10.26355/eurrev_202001_20029

Gao, M., Zhang, Z., Sun, J., Li, B., and Li, Y. (2022). The roles of circRNA-miRNA-mRNA networks in the development and treatment of osteoporosis. *Front Endocrinol (Lausanne)*, 13, 945310. doi:10.3389/fendo.2022.945310

Gao, X., Liu, S., and Liang, K. (2020). MicroRNA-19b-3p promotes cell proliferation and osteogenic differentiation of BMSCs by interacting with lncRNA H19. *BMC Med. Genet.* 21 (1), 11. doi:10.1186/s12881-020-0948-y

Gao, X., Xue, Y., and Yang, K. (2021). LINC00899 promotes osteogenic differentiation by targeting miR-374a and RUNX2 expression. *Exp. Ther. Med.* 22 (4), 1071. doi:10.3892/etm.2021.10505

Gao, Y., and Ge, W. (2018). The histone methyltransferase DOT1L inhibits osteoclastogenesis and protects against osteoporosis. *Cell Death Dis.* 9 (2), 33. doi:10.1038/s41419-017-0040-5

Gao, Y., Xiao, F., Wang, C., Wang, C., Cui, P., Zhang, X., et al. (2018). Long noncoding RNA MALAT1 promotes osterix expression to regulate osteogenic differentiation by targeting miRNA-143 in human bone marrow-derived mesenchymal stem cells. *J. Cell. Biochem.* 119 (8), 6986-6996. doi:10.1002/jcb.26907

Gu, H., Shi, S., Xiao, F., Huang, Z., Xu, J., Chen, G., et al. (2020). MiR-1-3p regulates the differentiation of mesenchymal stem cells to prevent osteoporosis by targeting secreted frizzled-related protein 1. *Bone* 137, 115444. doi:10.1016/j.bone.2020.115444

Gu, Z., Xie, D., Huang, C., Ding, R., Zhang, R., Li, Q., et al. (2020). MicroRNA-497 elevation or LRG1 knockdown promotes osteoblast proliferation and collagen synthesis in osteoporosis via TGF-β1/Smads signalling pathway. *J. Cell. Mol. Med.* 24 (21), 12619-12632. doi:10.1111/jcmm.15826

Guo, M., Liu, N., and Guo, Z. (2022). MiR-221-5p/Smad3 axis in osteoclastogenesis and its function: Potential therapeutic target for osteoporosis. *Steroids* 185, 109063. doi:10.1016/j.steroids.2022.109063

Guo, Q., Kang, H., Wang, J., Dong, Y., Peng, R., Zhao, H., et al. (2021). Inhibition of ACLY leads to suppression of osteoclast differentiation and function via regulation of histone acetylation. *J. Bone Miner. Res.* 36 (10), 2065-2080. doi:10.1002/jbmr.4399

Guo, Z., Xie, M., Zou, Y., Liang, Q., Liu, F., Su, J., et al. (2021). Circular RNA Hsa_circ_0006766 targets microRNA miR-4739 to regulate osteogenic differentiation of human bone marrow mesenchymal stem cells. *Bioengineered* 12 (1), 5679-5687. doi: 10.1080/21655979.2021.1967712

Han, Y., Zhang, K., Hong, Y., Wang, J., Liu, Q., Zhang, Z., et al. (2018). miR-342-3p promotes osteogenic differentiation via targeting ATF3. *FEBS Lett.* 592 (24), 4051-4065. doi:10.1002/1873-3468.13282

He, M., Lei, H., He, X., Liu, Y., Wang, A., Ren, Z., et al. (2022). METTL14 regulates osteogenesis of bone marrow mesenchymal stem cells via inducing autophagy through m6A/IGF2BPs/Beclin-1 signal axis. *Stem Cells Transl. Med.* 11 (9), 987-1001. doi:10.1093/stcltm/szac049

He, X., Zhu, L., An, L., and Zhang, J. (2020). MiR-143 inhibits osteoclastogenesis by targeting RANK and NF-κB and MAPK signaling pathways. *Curr. Mol. Pharmacol.* 13 (3), 224-232. doi:10.2174/1874467213666200116113945

He, Y., Chen, D., Guo, Q., Shi, P., You, C., and Feng, Y. (2021). MicroRNA-151a-3p functions in the regulation of osteoclast differentiation: significance to postmenopausal osteoporosis. *Clin. Interv. Aging* 216, 1357-1366. doi:10.2147/CIA.S289613

Hu, F., Jiang, C., Bu, G., Fu, Y., and Yu, Y. (2021). Silencing long noncoding RNA colon cancer-associated transcript-1 upregulates microRNA-34a-5p to promote proliferation and differentiation of osteoblasts in osteoporosis. *Cancer Gene Ther.* 28 (10-11), 1150-1161. doi:10.1038/s41417-020-00264-7

Hu, H., Zhao, C., Zhang, P., Liu, Y., Jiang, Y., Wu, E., et al. (2019). miR-26b modulates OA induced BMSC osteogenesis through regulating GSK3β/β-catenin pathway. *Exp. Mol. Pathol.* 107, 158-164. doi:10.1016/j.yexmp.2019.02.003

Hu, L. S., Guan, Z., Tang, C., Li, G., and Wen, J. (2022). Exosomes derived from microRNA-21 overexpressed adipose tissue-derived mesenchymal stem cells alleviate spine osteoporosis in ankylosing spondylitis mice. *J. Tissue Eng. Regen. Med.* 16 (7), 634-642. doi:10.1002/term.3304

Hu, L. C., Xie, X., Xue, H., Wang, T., Panayi, A. C., Lin, Z., et al. (2022). MiR-1224-5p modulates osteogenesis by coordinating osteoblast/osteoclast differentiation via the Rap1 signaling target ADCY2. *Exp. Mol. Med.* 54 (7), 961-972. doi:10.1038/s12276-022-00799-9

Hu, M., Xing, L., Zhang, L., Liu, F., Wang, S., Xie, Y., et al. (2022). NAP1L2 drives mesenchymal stem cell senescence and suppresses osteogenic differentiation. *Aging Cell* 21 (2), e13551. doi:10.1111/acel.13551

Hu, W. X., Li, H., and Jiang, J. Z. (2020). MiR-491-3p is down-regulated in postmenopausal osteoporosis and affects growth, differentiation and apoptosis of hFOB1.19 cells through targeting CTSS. *Folia Histochem. Cytobiol.* 58 (1), 9-16. doi:10.5603/FHC.a2020.0001

Hu, Z., Zhang, L., Wang, H., Wang, Y., Tan, Y., Dang, L., et al. (2020). Targeted silencing of miRNA-132-3p expression rescues disuse osteopenia by promoting mesenchymal stem cell osteogenic differentiation and osteogenesis in mice. *Stem Cell Res. Ther.* 11 (1), 58. doi:10.1186/s13287-020-1581-6

Huang, C., and Wang, Y. (2022). Downregulation of METTL14 improves postmenopausal osteoporosis via IGF2BP1 dependent posttranscriptional silencing of SMAD1. *Cell Death Dis*. 13 (11), 919. doi:10.1038/s41419-022-05362-y

Huang, M., Li, X., Zhou, C., Si, M., Zheng, H., Chen, L., et al. (2020). Noncoding RNA miR-205-5p mediates osteoporosis pathogenesis and osteoblast differentiation by regulating RUNX2. *J. Cell. Biochem.* 121 (10), 4196-4203. doi:10.1002/jcb.29599

Huang, M., Wang, Y., Wang, Z., Qin, Q., Zhang, H., Liu, S., et al. (2022). miR-134-5p inhibits osteoclastogenesis through a novel miR-134-5p/Itgb1/MAPK pathway. *J. Biol. Chem.* 298 (7), 102116. doi:10.1016/j.jbc.2022.102116

Huang, Y., Ren, K., Yao, T., Zhu, H., Xu, Y., Ye, H., et al. (2020). MicroRNA-25-3p regulates osteoclasts through nuclear factor I X. *Biochem. Biophys. Res. Commun.* 522 (1), 74-80. doi:10.1016/j.bbrc.2019.11.043

Ji, H., Cui, X., Yang, Y., and Zhou, X. (2021). CircRNA hsa_circ_0006215 promotes osteogenic differentiation of BMSCs and enhances osteogenesis-angiogenesis coupling by competitively binding to miR-942-5p and regulating RUNX2 and VEGF. *Aging (Albany NY)* 13 (7), 10275-10288. doi:10.18632/aging.202791

Jiang, Y., Wu, W., Jiao, G., Chen, Y., and Liu, H. (2019). LncRNA SNHG1 modulates p38 MAPK pathway through Nedd4 and thus inhibits osteogenic differentiation of bone marrow mesenchymal stem cells. *Life Sci*. 228, 208-214. doi:10.1016/j.lfs.2019.05.002

Jin, C., Jia, L., Tang, Z., and Zheng, Y. (2020). Long non-coding RNA MIR22HG promotes osteogenic differentiation of bone marrow mesenchymal stem cells via PTEN/ AKT pathway. *Cell Death Dis*. 11 (7), 601. doi:10.1038/s41419-020-02813-2

Jin, S. L., Bai, Y. M., Zhao, B. Y., Wang, Q. H., and Zhang, H. S. (2020). Silencing of miR-330-5p stimulates osteogenesis in bone marrow mesenchymal stem cells and inhibits bone loss in osteoporosis by activating Bgn-mediated BMP/Smad pathway. *Eur. Rev. Med. Pharmacol. Sci.* 24 (8), 4095-4102. doi:10.26355/eurrev_202004_20987

Jing, H., Su, X., Gao, B., Shuai, Y., Chen, J., Deng, Z., et al. (2018). Epigenetic inhibition of Wnt pathway suppresses osteogenic differentiation of BMSCs during osteoporosis. *Cell Death Dis.* 9 (2), 176. doi:10.1038/s41419-017-0231-0

Karimi, Z., Seyedjafari, E., Khojasteh, A., Hashemi, S. M., Kazemi, B., and Mohammadi-Yeganeh, S. (2020). MicroRNA-218 competes with differentiation media in the induction of osteogenic differentiation of mesenchymal stem cell by regulating β-catenin inhibitors. *Mol. Biol. Rep.* 47 (11), 8451-8463. doi:10.1007/s11033-020-05885-7

Kong, Y., Nie, Z. K., Li, F., Guo, H. M., Yang, X. L., and Ding, S. F. (2019). MiR-320a was highly expressed in postmenopausal osteoporosis and acts as a negative regulator in MC3T3E1 cells by reducing MAP9 and inhibiting PI3K/AKT signaling pathway. *Exp. Mol. Pathol.* 110, 104282. doi:10.1016/j.yexmp.2019.104282

Kou, J., Zheng, X., Guo, J., Liu, Y., and Liu, X. (2020). MicroRNA-218-5p relieves postmenopausal osteoporosis through promoting the osteoblast differentiation of bone marrow mesenchymal stem cells. *J. Cell. Biochem.* 121 (2), 1216-1226. doi:10.1002/jcb.29355

Kureel, J., John, A. A., Prakash, R., and Singh, D. (2018). MiR 376c inhibits osteoblastogenesis by targeting Wnt3 and ARF-GEF-1 -facilitated augmentation of beta-catenin transactivation. *J. Cell Biochem.* 119 (4), 3293-3303. doi:10.1002/jcb.26490

Li, B., Wang, J., Xu, F., Wang, Q., Liu, Q., Wang, G., et al. (2023). LncRNA RAD51-AS1 regulates human bone marrow mesenchymal stem cells via interaction with YBX1 to ameliorate osteoporosis. *Stem Cell Rev. Rep.* 19 (1), 170-187. doi:10.1007/s12015-022-10408-x

Li, B., Wu, P., Fu, W., Xiong, Y., Zhang, L., Gao, Y., et al. (2019). The role and mechanism of miRNA-1224 in the Polygonatum sibiricum polysaccharide regulation of bone marrow-derived macrophages to osteoclast differentiation. *Rejuvenation Res.* 22 (5), 420-430. doi:10.1089/rej.2018.2126

Li, B., Zhao, J., Ma, J. X., Li, G. M., Zhang, Y., Xing, G. S., et al. (2018). Overexpression of DNMT1 leads to hypermethylation of H19 promoter and inhibition of Erk signaling pathway in disuse osteoporosis. *Bone* 111, 82-91. doi:10.1016/j.bone.2018.03.017

Li, D. J., Liu, G. Q., and Xu, X. J. (2020). Silence of lncRNA BCAR4 alleviates the deterioration of osteoporosis. *Eur. Rev. Med. Pharmacol. Sci.* 24 (11), 5905-5913. doi:10.26355/eurrev_202006_21483

Li, D., Tian, Y., Yin, C., Huai, Y., Zhao, Y., Su, P., et al. (2019). Silencing of lncRNA AK045490 promotes osteoblast differentiation and bone formation via β-Catenin/TCF1/Runx2 signaling axis. *Int. J. Mol. Sci.* 2019, 20 (24), 6229. doi:10.3390/ijms20246229

Li, D., Yuan, Q., Xiong, L., Li, A., and Xia, Y. (2021). The miR-4739/DLX3 axis modulates bone marrow-derived mesenchymal stem cell (BMSC) osteogenesis affecting osteoporosis progression. *Front. Endocrinol. (Lausanne)* 12, 703167. doi:10.3389/fendo.2021.703167

Li, F., Wu, H., Zou, G., Cang, D., and Shen, Y. (2021). Circular RNA_0062582 promotes osteogenic differentiation of human bone marrow mesenchymal stem cells via regulation of microRNA-145/CBFB axis. *Bioengineered* 12 (1), 1952-1963. doi:10.1080/21655979.2021.1921553

Li, H., Li, T., Fan, J., Li, T., Fan, L., Wang, S., et al. (2015). miR-216a rescues dexamethasone suppression of osteogenesis, promotes osteoblast differentiation and enhances bone formation, by regulating c-Cbl-mediated PI3K/AKT pathway. *Cell Death Differ*. 22 (12), 1935-1945. doi:10.1038/cdd.2015.99

Li, J. P., Zhuang, H. T., Xin, M. Y., and Zhou, Y. L. (2017). MiR-214 inhibits human mesenchymal stem cells differentiating into osteoblasts through targeting β-catenin. *Eur. Rev. Med. Pharmacol. Sci.* 21 (21), 4777-4783

Li, J. Y., Wei, X., Sun, Q., Zhao, X. Q., Zheng, C. Y., Bai, C. X., et al. (2019). MicroRNA-449b-5p promotes the progression of osteoporosis by inhibiting osteogenic differentiation of BMSCs via targeting Satb2. *Eur. Rev. Med. Pharmacol. Sci.* 23 (15), 6394-6403. doi:10.26355/eurrev_201908_18519

Li, J., Feng, Z., Chen, L., Wang, X., and Deng, H. (2016). MicroRNA-335-5p inhibits osteoblast apoptosis induced by high glucose. *Mol. Med. Rep.* 13 (5), 4108-4112. doi:10.3892/mmr.2016.4994

Li, J., He, C., Tong, W., Zou, Y., Li, D., Zhang, C., et al. (2015). Tanshinone IIA blocks dexamethasone-induced apoptosis in osteoblasts through inhibiting Nox4-derived ROS production. *Int. J. Clin. Exp. Pathol.* 8 (10), 13695-13706.

Li, K., Chen, S., Cai, P., Chen, K., Li, L., Yang, X., et al. (2020). MiRNA-483-5p is involved in the pathogenesis of osteoporosis by promoting osteoclast differentiation. *Mol. Cell. Probes* 49, 101479. doi:10.1016/j.mcp.2019.101479

Li, L. Y., Wang, X. L., Wang, G. S., and Zhao, H. Y. (2019). MiR-373 promotes the osteogenic differentiation of BMSCs from the estrogen deficiency induced osteoporosis. *Eur. Rev. Med. Pharmacol. Sci.* 23 (17), 7247-7255. doi:10.26355/eurrev_201909_18827

Li, L. P., Wang, H., Chen, X., Li, X., Wang, G., Jie, Z., et al. (2021). Oxidative stress-induced hypermethylation of KLF5 promoter mediated by DNMT3B impairs osteogenesis by diminishing the interaction with β-catenin. *Antioxid. Redox Signal.* 35 (1), 1-20. doi:10.1089/ars.2020.8200

Li, L., Zhou, X., Zhang, J. T., Liu, A. F., Zhang, C., Han, J. C., et al. (2021). Exosomal miR-186 derived from BMSCs promote osteogenesis through hippo signaling pathway in postmenopausal osteoporosis. *J. Orthop. Surg. Res.* 16 (1), 23. doi:10.1186/s13018-020-02160-0

Li, M., Cong, R., Yang, L., Yang, L., Zhang, Y., and Fu, Q. (2020). A novel lncRNA LNC_000052 leads to the dysfunction of osteoporotic BMSCs via the miR-96-5p-PIK3R1 axis. *Cell Death Dis*. 11 (9), 795. doi:10.1038/s41419-020-03006-7

Li, M., Li, C., Zheng, H., Zhou, Z., Yang, W., Gong, Y., et al. (2022). CircRNA_0001795 sponges miRNA-339-5p to regulate yes-associated protein 1 expression and attenuate osteoporosis progression. *Bioengineered* 13 (2), 2803-2815. doi:10.1080/21655979.2021.2022074

Li, R., Shi, T. T., Wang, Q., and Zhang, Y. X. (2022). Elevated lncRNA MIAT in peripheral blood mononuclear cells contributes to post-menopausal osteoporosis. *Aging (Albany NY)* 14 (7), 3143-3154. doi:10.18632/aging.204001

Li, W., Chen, Z., Cai, C., Li, G., Wang, X., and Shi, Z. (2020). MicroRNA-505 is involved in the regulation of osteogenic differentiation of MC3T3-E1 cells partially by targeting RUNX2. *J. Orthop. Surg. Res.* 15 (1), 143. doi:10.1186/s13018-020-01645-2

Li, X., Wu, J., Liu, S., Zhang, K., Miao, X., Li, J., et al. (2019). miR-384-5p targets Gli2 and negatively regulates age-related osteogenic differentiation of rat bone marrow mesenchymal stem cells. *Stem Cells Dev.* 28 (12), 791-798. doi:10.1089/scd.2019.0044

Li, Y., Feng, C., Gao, M., Jin, M., Liu, T., Yuan, Y., et al. (2019). MicroRNA-92b-5p modulates melatonin-mediated osteogenic differentiation of bone marrow mesenchymal stem cells by targeting ICAM-1. *J. Cell. Mol. Med.* 23 (9), 6140-6153. doi:10.1111/jcmm.14490

Li, Z. H., Hu, H., Zhang, X. Y., Liu, G. D., Ran, B., Zhang, P. G., et al. (2020). MiR-291a-3p regulates the BMSCs differentiation via targeting DKK1 in dexamethasone-induced osteoporosis. *Kaohsiung J. Med. Sci.* 36 (1), 35-42. doi:10.1002/kjm2.12134

Li, Z., Zhang, W., and Huang, Y. (2018). MiRNA-133a is involved in the regulation of postmenopausal osteoporosis through promoting osteoclast differentiation. *Acta Biochim. Biophys. Sin. (Shanghai)* 50 (3), 273-280. doi: 10.1093/abbs/gmy006. PMID: 29425279

Li, Z., Zhao, H., Chu, S., Liu, X., Qu, X., Li, J., et al. (2020). miR-124-3p promotes BMSC osteogenesis via suppressing the GSK-3β/β-catenin signaling pathway in diabetic osteoporosis rats. *In Vitro Cell. Dev. Biol. Anim.* 56 (9), 723-734. doi: 10.1007/s11626-020-00502-0

Lian, W. S., Ko, J. Y., Chen, Y. S., Ke, H. J., Hsieh, C. K., Kuo, C. W., et al. (2019). MicroRNA-29a represses osteoclast formation and protects against osteoporosis by regulating PCAF-mediated RANKL and CXCL12. *Cell Death Dis*. 10 (10), 705. doi:10.1038/s41419-019-1942-1

Lian, W. S., Wu, R. W., Chen, Y. S., Ko, J. Y., Wang, S. Y., Jahr, H., et al. (2021a). MicroRNA-29a mitigates osteoblast senescence and counteracts bone loss through oxidation resistance-1 control of FoxO3 methylation. *Antioxidants (Basel)* 10 (8), 1248. doi:10.3390/antiox10081248

Lian, W. S., Wu, R. W., Chen, Y. S., Ko, J. Y., Wang, S. Y., Jahr, H., et al. (2021b). MicroRNA-29a in osteoblasts represses high-fat diet-mediated osteoporosis and body adiposis through targeting leptin. *Int. J. Mol. Sci.* 22 (17), 9135. doi:10.3390/ijms22179135

Lin, C., Yu, S., Jin, R., Xiao, Y., Pan, M., Pei, F., et al. (2019). Circulating miR-338 Cluster activities on osteoblast differentiation: Potential Diagnostic and Therapeutic Targets for Postmenopausal Osteoporosis. *Theranostics* 9 (13), 3780-3797. doi:10.7150/thno.34493

Lin, J. C., Liu, Z. G., Yu, B., and Zhang, X. R. (2018). MicroRNA-874 targeting SUFU involves in osteoblast proliferation and differentiation in osteoporosis rats through the Hedgehog signaling pathway. *Biochem. Biophys. Res. Commun.* 506 (1), 194-203. doi:10.1016/j.bbrc.2018.09.187

Lin, Y. P., Liao, L. M., Liu, Q. H., Ni, Y., Zhong, Y., and Yu, S. (2021). MiRNA-128-3p induces osteogenic differentiation of bone marrow mesenchymal stem cells via activating the Wnt3a signaling. *Eur. Rev. Med. Pharmacol. Sci.* 25 (3), 1225-1232. doi:10.26355/eurrev_202102_24826

Ling, L., Hu, H. L., Liu, K. Y., Ram, Y. I., Gao, J. L., and Cao, Y. M. (2019). Long noncoding RNA MIRG induces osteoclastogenesis and bone resorption in osteoporosis through negative regulation of miR-1897. *Eur. Rev. Med. Pharmacol. Sci.* 23 (23), 10195-10203. doi:10.26355/eurrev_201912_19654

Liu, D., Lin, Z., Huang, Y., and Qiu, M. (2022). Role of microRNA-19b-3p on osteoporosis after experimental spinal cord injury in rats. *Arch. Biochem. Biophys.* 719, 109134. doi:10.1016/j.abb.2022.109134

Liu, F., Liang, Y., and Lin, X. (2022). MiR-151b inhibits osteoblast differentiation via downregulating Msx2. *Connect Tissue Res*. 63 (2), 112-123. doi:10.1080/03008207.2021.1882442

Liu, H. P., Hao, D. J., Wang, X. D., Hu, H. M., Li, Y. B., and Dong, X. H. (2019). MiR-30a-3p promotes ovariectomy-induced osteoporosis in rats via targeting SFRP1. *Eur. Rev. Med. Pharmacol. Sci.* 23 (22), 9754-9760. doi:10.26355/eurrev_201911_19538

Liu, H., Liu, Q., Wu, X. P., He, H. B., and Fu, L. (2018). MiR-96 regulates bone metabolism by targeting osterix. *Clin. Exp. Pharmacol. Physiol.* 45 (6), 602-613. doi:10.1111/1440-1681.12912

Liu, H., Sun, Q., Wan, C., Li, L., Zhang, L., and Chen, Z. (2014). MicroRNA-338-3p regulates osteogenic differentiation of mouse bone marrow stromal stem cells by targeting Runx2 and Fgfr2. *J. Cell. Physiol.* 229 (10), 1494-1502. doi:10.1002/jcp.24591

Liu, H., Yue, X., and Zhang, G. (2021b). Downregulation of miR‑146a inhibits osteoporosis in the jaws of ovariectomized rats by regulating the Wnt/β‑catenin signaling pathway. *Int. J. Mol. Med.* 47 (3), 6. doi:10.3892/ijmm.2020.4839

Liu, J., Chen, M., Ma, L., Dang, X., and Du, G. (2021). piRNA-36741 regulates BMP2-mediated osteoblast differentiation via METTL3 controlled m6A modification. *Aging (Albany NY)* 13 (19), 23361-23375. doi:10.18632/aging.203630

Liu, K., Jing, Y., Zhang, W., Fu, X., Zhao, H., Zhou, X., et al. (2017). Silencing miR-106b accelerates osteogenesis of mesenchymal stem cells and rescues against glucocorticoid-induced osteoporosis by targeting BMP2. *Bone* 97, 130-138. doi:10.1016/j.bone.2017.01.014

Liu, S., Yang, X., Zhong, X., Li, L., and Zhang, X. (2021). Involvement of miR-337 in high glucose-suppressed osteogenic differentiation in bone marrow mesenchymal stem cells via negative regulation of Rap1A. *In Vitro Cell. Dev. Biol. Anim.* 57 (3), 350-358. doi:10.1007/s11626-021-00553-x

Liu, T. J., and Guo, J. L. (2020). Overexpression of microRNA-141 inhibits osteoporosis in the jawbones of ovariectomized rats by regulating the Wnt/β-catenin pathway. *Arch. Oral Biol.* 113, 104713. doi:10.1016/j.archoralbio.2020.104713

Liu, T., Zheng, X., Wang, C., Wang, C., Jiang, S., Li, B., et al. (2021). The m6A "reader" YTHDF1 promotes osteogenesis of bone marrow mesenchymal stem cells through translational control of ZNF839. *Cell Death Dis*. 12 (11), 1078. doi:10.1038/s41419-021-04312-4

Liu, W., Li, G., Li, J., and Chen, W. (2022). Long noncoding RNA TRG-AS1 protects against glucocorticoid-induced osteoporosis in a rat model by regulating miR-802-mediated CAB39/AMPK/SIRT-1/NF-κB axis. Hum. Cell 35 (5), 1424-1439. doi:10.1007/s13577-022-00741-1

Liu, X. D., Cai, F., Liu, L., Zhang, Y., and Yang, A. L. (2015). MicroRNA-210 is involved in the regulation of postmenopausal osteoporosis through promotion of VEGF expression and osteoblast differentiation. Biol. Chem. 396 (4), 339-347. doi:10.1515/hsz-2014-0268

Liu, X., Chen, M., Liu, Q., Li, G., Yang, P., and Zhang, G. (2022). LncRNA PTCSC3 is upregulated in osteoporosis and negatively regulates osteoblast apoptosis. *BMC Med. Genomics* 15 (1), 57. doi:10.1186/s12920-022-01182-3

Liu, Z. H., Qi, D. D., Li, X., Zhang, S. Q., Zhao, Y., Fu, L. X., et al. (2021). LncRNA SNHG14 promotes osteogenic differentiation of human bone marrow-derived mesenchymal stem cells via regulating miR-185-5p/WISP2 axis. *J. Biol. Regul. Homeost. Agents* 35 (2), 605-615. doi:10.23812/20-391-A

Liu, Z. Z., Hong, C. G., Hu, W. B., Chen, M. L., Duan, R., Li, H. M., et al. (2021). Autophagy receptor OPTN (optineurin) regulates mesenchymal stem cell fate and bone-fat balance during aging by clearing FABP3. *Autophagy* 17 (10), 2766-2782. doi:10.1080/15548627.2020.1839286

Lu, X. D., Han, W. X., and Liu, Y. X. (2019). Suppression of miR-451a accelerates osteogenic differentiation and inhibits bone loss via Bmp6 signaling during osteoporosis. *Biomed. Pharmacother.* 120, 109378. doi:10.1016/j.biopha.2019.109378

Lu, X. Z., Yang, Z. H., Zhang, H. J., Zhu, L. L., Mao, X. L., and Yuan, Y. (2017). MiR-214 protects MC3T3-E1 osteoblasts against H2O2-induced apoptosis by suppressing oxidative stress and targeting ATF4. *Eur. Rev. Med. Pharmacol. Sci.* 21 (21), 4762-4770

Lu, X., Zhang, Y., Zheng, Y., and Chen, B. (2021). The miRNA-15b/USP7/KDM6B axis engages in the initiation of osteoporosis by modulating osteoblast differentiation and autophagy. *J. Cell. Mol. Med.* 25 (4), 2069-2081. doi:10.1111/jcmm.16139

Lu, Z., Wang, D., Wang, X., Zou, J., Sun, J., and Bi, Z. (2021). MiR-206 regulates the progression of osteoporosis via targeting HDAC4. *Eur. J. Med. Res.* 26 (1), 8. doi:10.1186/s40001-021-00480-3

Luo, B., Yang, J. F., Wang, Y. H., Qu, G. B., Hao, P. D., Zeng, Z. J., et al. (2019). MicroRNA-579-3p promotes the progression of osteoporosis by inhibiting osteogenic differentiation of mesenchymal stem cells through regulating Sirt1. *Eur. Rev. Med. Pharmacol. Sci.* 23 (16), 6791-6799. doi:10.26355/eurrev_201908_18717

Luo, B., Yang, J., Yuan, Y., Hao, P., and Cheng, X. (2020). MicroRNA-142 regulates osteoblast differentiation and apoptosis of mouse pre-osteoblast cells by targeting bone morphogenetic protein 2. *FEBS Open Bio* 10 (9), 1793-1801. doi:10.1002/2211-5463.12929

Lv, H., Sun, Y., and Zhang, Y. (2015). MiR-133 is involved in estrogen deficiency-induced osteoporosis through modulating osteogenic differentiation of mesenchymal stem cells. *Med. Sci. Monit.* 21, 1527-1534. doi:10.12659/MSM.894323

Lv, R., Pan, X., Song, L., Sun, Q., Guo, C., Zou, S., et al. (2019). MicroRNA-200a-3p accelerates the progression of osteoporosis by targeting glutaminase to inhibit osteogenic differentiation of bone marrow mesenchymal stem cells. *Biomed. Pharmacother.* 116, 108960. doi:10.1016/j.biopha.2019.108960

Ma, C., Gao, J., Liang, J., Dai, W., Wang, Z., Xia, M., et al. (2021). HDAC6 inactivates Runx2 promoter to block osteogenesis of bone marrow stromal cells in age-related bone loss of mice. *Stem Cell Res. Ther.* 12 (1), 484. doi:10.1186/s13287-021-02545-w

Ma, Q. Q., Lin, L., Yao, Q., Yang, J., Hu, Y., and Yu, J. B. (2018). Reduced CpG island methylation of the TBC1D8 gene may predict risk for osteoporosis in Chinese postmenopausal women. *Oncotarget* 11 (47), 4448-4456. doi:10.18632/oncotarget.24065

Ma, T. L., Zhu, P., Ke, Z. R., Chen, J. X., Hu, Y. H., and Xie, J. (2022). Focusing on OB-OC-MΦ Axis and miR-23a to explore the pathogenesis and treatment strategy of osteoporosis. *Front. Endocrinol. (Lausanne)* 13, 891313. doi:10.3389/fendo.2022.891313

Ma, Y., Liu, H., Lu, X., Song, C., Cheng, Y., Wang, Y., et al. (2022). Exploring the potential mechanism of artemisinin and its derivatives in the treatment of osteoporosis based on network pharmacology and molecular docking. *Comput. Math. Methods Med.* 2022, 3976062. doi:10.1155/2022/3976062

Mao, J. H., Sui, Y. X., Ao, S., Wang, Y., Liu, Y., and Leng, H. (2019). miR-140-3p exhibits repressive functions on preosteoblast viability and differentiation by downregulating MCF2L in osteoporosis. *In Vitro Cell Dev. Biol. Anim.* 56 (1), 49-58. doi:10.1007/s11626-019-00405-9

Mao, Z., Zhu, Y., Hao, W., Chu, C., and Su, H. (2019). MicroRNA-155 inhibition up-regulates LEPR to inhibit osteoclast activation and bone resorption via activation of AMPK in alendronate-treated osteoporotic mice. *IUBMB Life* 71 (12), 1916-1928. doi:10.1002/iub.2131

Mei, B., Wang, Y., Ye, W., Huang, H., Zhou, Q., Chen, Y., et al. (2019). LncRNA ZBTB40-IT1 modulated by osteoporosis GWAS risk SNPs suppresses osteogenesis. Hum Genet. 138 (2), 151-166. doi:10.1007/s00439-019-01969-y

Miao, M., Zhang, Y., Wang, X., Lei, S., Huang, X., Qin, L., et al. (2022). The miRNA-144-5p/IRS1/AKT axis regulates the migration, proliferation, and mineralization of osteoblasts: A mechanism of bone repair in diabetic osteoporosis. *Cell Biol. Int.* 46 (12), 2220-2231. doi:10.1002/cbin.11913

Mori, K., Kitazawa, R., Kondo, T., Mori, M., Hamada, Y., Nishida, M., et al. (2014). Diabetic osteopenia by decreased β-catenin signaling is partly induced by epigenetic derepression of sFRP-4 gene. *PLoS One* 9 (7), e102797. doi:10.1371/journal.pone.0102797

Mulati, M., Kobayashi, Y., Takahashi, A., Numata, H., Saito, M., Hiraoka, Y., et al. (2020). The long noncoding RNA Crnde regulates osteoblast proliferation through the Wnt/β-catenin signaling pathway in mice. *Bone* 130, 115076. doi:10.1016/j.bone.2019.115076

Najafova, Z., Liu, P., Wegwitz, F., Ahmad, M., Tamon, L., Kosinsky, R. L., et al. (2021). RNF40 exerts stage-dependent functions in differentiating osteoblasts and is essential for bone cell crosstalk. *Cell Death Differ.* 28 (2), 700-714. doi:10.1038/s41418-020-00614-w

Ouyang, X., Ding, Y., Yu, L., Xin, F., and Yang, X. (2022). LncRNA TUG regulates osteogenic differentiation of bone marrow mesenchymal stem cells via miRNA-204/SIRT 1. *J. Musculoskelet. Neuronal. Interact.* 22 (3), 401-410.

Panach, L., Pineda, B., Mifsut, D., Tarín, J. J., Cano, A., and García-Pérez, M. Á. (2016). The role of CD40 and CD40L in bone mineral density and in osteoporosis risk: A genetic and functional study. *Bone* 83, 94-103. doi:10.1016/j.bone.2015.11.002

Peng, S., Gao, Y., Shi, S., Zhao, D., Cao, H., Fu, T., et al. (2022). LncRNA-AK137033 inhibits the osteogenic potential of adipose-derived stem cells in diabetic osteoporosis by regulating Wnt signaling pathway via DNA methylation. *Cell Prolif.* 55 (1), e13174. doi:10.1111/cpr.13174

Peng, S., Shi, S., Tao, G., Li, Y., Xiao, D., Wang, L., et al. (2021). JKAMP inhibits the osteogenic capacity of adipose-derived stem cells in diabetic osteoporosis by modulating the Wnt signaling pathway through intragenic DNA methylation. *Stem Cell Res. Ther.* 12 (1), 120. doi:10.1186/s13287-021-02163-6

Pucci, S., Greggi, C., Polidoro, C., Piro, M. C., Celi, M., Feola, M., et al. (2019). Clusterin silencing restores myoblasts viability and down modulates the inflammatory process in osteoporotic disease. *J. Transl. Med.* 17 (1), 118. doi:10.1186/s12967-019-1868-5

Qi, Q., Wang, Y., Wang, X., Yang, J., Xie, Y., Zhou, J., et al. (2019). Histone demethylase KDM4A regulates adipogenic and osteogenic differentiation via epigenetic regulation of C/EBPα and canonical Wnt signaling. *Cell Mol. Life Sci.* 77 (12), 2407-2421. doi:10.1007/s00018-019-03289-w

Qi, X. B., Jia, B., Wang, W., Xu, G. H., Guo, J. C., Li, X., et al. (2019). Role of miR-199a-5p in osteoblast differentiation by targeting TET2. *Gene* 726, 144193. doi:10.1016/j.gene.2019.144193

Qiao, L., Liu, D., Li, C. G., and Wang, Y. J. (2018). MiR-203 is essential for the shift from osteogenic differentiation to adipogenic differentiation of mesenchymal stem cells in postmenopausal osteoporosis. *Eur. Rev. Med. Pharmacol. Sci.* 22 (18), 5804-5814. doi:10.26355/eurrev_201809_15906

Qin, X. B., Wen, K., Wu, X. X., and Yao, Z. J. (2021). MiR-183 regulates the differentiation of osteoblasts in the development of osteoporosis by targeting Smad4. *Acta Histochem.* 123 (7), 151786. doi:10.1016/j.acthis.2021.151786

Qiu, J., Huang, G., Na, N., and Chen, L. (2018). MicroRNA-214-5p/TGF-β/Smad2 signaling alters adipogenic differentiation of bone marrow stem cells in postmenopausal osteoporosis. *Mol. Med. Rep.* 17 (5), 6301-6310. doi:10.3892/mmr.2018.8713

Qu, B., He, J., Zeng, Z., Yang, H., Liu, Z., Cao, Z., et al. (2020). MiR-155 inhibition alleviates suppression of osteoblastic differentiation by high glucose and free fatty acids in human bone marrow stromal cells by upregulating SIRT1. *Pflugers Arch.* 472 (4), 473-480. doi:10.1007/s00424-020-02372-7

Qu, B., Xia, X., Yan, M., Gong, K., Deng, S., Huang, G., et al. (2015). miR-218 is involved in the negative regulation of osteoclastogenesis and bone resorption by partial suppression of p38MAPK-c-Fos-NFATc1 signaling: Potential role for osteopenic diseases. *Exp. Cell Res.* 338 (1), 89-96. doi:10.1016/j.yexcr.2015.07.023

Raje, M. M., and Ashma, R. (2019). Epigenetic regulation of BMP2 gene in osteoporosis: A DNA methylation study. *Mol. Biol. Rep.* 46 (2), 1667-1674. doi:10.1007/s11033-019-04615-y

Ramírez-Salazar, E. G., Almeraya, E. V., López-Perez, T. V., Patiño, N., Salmeron, J., and Velázquez-Cruz, R. (2020). MicroRNA-548-3p overexpression inhibits proliferation, migration and invasion in osteoblast-like cells by targeting STAT1 and MAFB. *J. Biochem.* 168 (3), 203-211. doi:10.1093/jb/mvaa033

Ren, L. R., Yao, R. B., Wang, S. Y., Gong, X. D., Xu, J. T., and Yang, K. S. (2021). MiR-27a-3p promotes the osteogenic differentiation by activating CRY2/ERK1/2 axis. *Mol. Med.* 27 (1), 43. doi:10.1186/s10020-021-00303-5

Reppe, S., Lien, T. G., Hsu, Y. H., Gautvik, V. T., Olstad, O. K., Yu, R., et al. (2017). Distinct DNA methylation profiles in bone and blood of osteoporotic and healthy postmenopausal women. *Epigenetics* 12 (8), 674-687. doi:10.1080/15592294.2017.1345832

Reppe, S., Noer, A., Grimholt, R. M., Halldorsson, B. V., Medina-Gomez, C., Gautvik, V. T., et al. (2015). Methylation of bone SOST, its mRNA, and serum sclerostin levels correlate strongly with fracture risk in postmenopausal women. *J. Bone Miner. Res.* 30 (2), 249-256. doi:10.1002/jbmr.2342

Schroeder, T. M., and Westendorf, J. J. (2005). Histone deacetylase inhibitors promote osteoblast maturation. *J Bone Miner. Res.* 20 (12), 2254-2263. doi:10.1359/JBMR.050813

Shan, Y., Wang, L., Li, G., Shen, G., Zhang, P., and Xu, Y. (2019). Methylation of bone SOST impairs SP7, RUNX2, and ERα transactivation in patients with postmenopausal osteoporosis. *Biochem. Cell Biol.* 97 (4), 369-374. doi:10.1139/bcb-2018-0170

Shangguan, Y., Li, X., Qin, J., Wen, Y., Wang, H., and Chen, L. (2022). Positive programming of the GC-IGF1 axis mediates adult osteoporosis susceptibility in male offspring rats induced by prenatal dexamethasone exposure. *Biochem. Pharmacol.* 206, 115264. doi:10.1016/j.bcp.2022.115264

Shao, B., Fu, X., Yu, Y., and Yang, D. (2018). Regulatory effects of miRNA‑181a on FasL expression in bone marrow mesenchymal stem cells and its effect on CD4+T lymphocyte apoptosis in estrogen deficiency‑induced osteoporosis. *Mol. Med. Rep.* 18 (1), 920-930. doi:10.3892/mmr.2018.9026

Shao, Y., Hu, X., and Wu, X. (2021). LncRNA X inactive-specific transcript promotes osteoclast differentiation through Tgif2 by acting as a ceRNA of miR-590-3p in a murine model. *Regen. Med.* 16 (7), 643-653. doi:10.2217/rme-2020-0174

Shen, G. S., Zhou, H. B., Zhang, H., Chen, B., Liu, Z. P., Yuan, Y., et al. (2018). The GDF11-FTO-PPARγ axis controls the shift of osteoporotic MSC fate to adipocyte and inhibits bone formation during osteoporosis. *Biochim. Biophys. Acta Mol. Basis Dis.* 1864 (12), 3644-3654. doi:10.1016/j.bbadis.2018.09.015

Shen, J. J., Zhang, C. H., Chen, Z. W., Wang, Z. X., Yang, D. C., Zhang, F. L., et al. (2019). LncRNA HOTAIR inhibited osteogenic differentiation of BMSCs by regulating Wnt/β-catenin pathway. *Eur. Rev. Med. Pharmacol. Sci.* 23 (17), 7232-7246. doi:10.26355/eurrev_201909_18826

Shi, Z. L., Zhang, H., Fan, Z. Y., Ma, W., Song, Y. Z., Li, M., et al. (2020). Long noncoding RNA LINC00314 facilitates osteogenic differentiation of adipose-derived stem cells through the hsa-miR-129-5p/GRM5 axis via the Wnt signaling pathway. *Stem Cell Res. Ther.* 11 (1), 240. doi:10.1186/s13287-020-01754-z

Shi, Z., Zhong, Q., Chen, Y., and Luo, X. (2022). Long noncoding RNA ZBTB40-IT1 regulates bone mass by directing the differentiation of human bone marrow mesenchymal stromal cells via the microRNA-514a-3p/FOXO4 axis. *Hum. Cell* 35 (5), 1408-1423. doi:10.1007/s13577-022-00730-4

Song, C., Guo, Y., Chen, F., and Liu, W. (2022). lncRNA MALAT1 promotes osteogenic differentiation through the miR-217/AKT3 axis: A possible strategy to alleviate osteoporosis. *J. Gene Med.* 24 (6), e3409. doi:10.1002/jgm.3409

Song, Q., Zhong, L., Chen, C., Tang, Z., Liu, H., Zhou, Y., et al. (2015). miR-21 synergizes with BMP9 in osteogenic differentiation by activating the BMP9/Smad signaling pathway in murine multilineage cells. *Int. J. Mol. Med.* 36 (6), 1497-1506. doi:10.3892/ijmm.2015.2363

Su, Y., Meng, X., Wang, W., Gu, G., and Chen, Y. (2021). LncRNA HOTAIR regulates fracture healing in osteoporotic rats through inhibition on MiR-17-5p. *Minerva Med*. 112 (4), 525-527. doi:10.23736/S0026-4806.19.06246-3

Sun, J., Ermann, J., Niu, N., Yan, G., Yang, Y., Shi, Y., et al. (2018). Histone demethylase LSD1 regulates bone mass by controlling WNT7B and BMP2 signaling in osteoblasts. *Bone Res*. 6, 14. doi:10.1038/s41413-018-0015-x

Sun, L., Lian, J. X., and Meng, S. (2019). MiR-125a-5p promotes osteoclastogenesis by targeting TNFRSF1B. *Cell. Mol. Biol. Lett.* 24, 23. doi:10.1186/s11658-019-0146-0

Sun, M., Hu, L., Wang, S., Huang, T., Zhang, M., Yang, M., et al. (2019). Circulating microRNA-19b identified from osteoporotic vertebral compression fracture patients increases bone formation. *J. Bone Miner. Res.* 35(2), 306-316. doi:10.1002/jbmr.3892

Sun, T., Li, C. T., Xiong, L., Ning, Z., Leung, F., Peng, S., et al. (2017). miR-375-3p negatively regulates osteogenesis by targeting and decreasing the expression levels of LRP5 and β-catenin. *PLoS One* 12 (2), e0171281. doi:10.1371/journal.pone.0171281

Sun, Y., Cai, M., Zhong, J., Yang, L., Xiao, J., Jin, F., et al. (2019). The long noncoding RNA lnc-ob1 facilitates bone formation by upregulating Osterix in osteoblasts. Nat. Metab. 1 (4), 485-496. doi:10.1038/s42255-019-0053-8

Sun, Y., Wang, X., Chen, G., Song, C., Ma, X., Fu, Y., et al. (2020). miRNA-187-5p regulates osteoblastic differentiation of bone marrow mesenchymal stem cells in mice by targeting ICAM1. *Biomed. Res. Int.* 2020, 6139469. doi:10.1155/2020/6139469

Sun, Z., Wang, H., Wang, Y., Yuan, G., Yu, X., Jiang, H., et al. (2021). MiR-103-3p targets the m^6^A methyltransferase METTL14 to inhibit osteoblastic bone formation. *Aging Cell* 20 (2), e13298. doi: 10.1111/acel.13298

Tan, H., Wang, Y., Zou, Z., Xing, Y., Shi, Z., Wang, K., et al. (2022). Facilitative role of circPVT1 in osteogenic differentiation potentials of bone marrow mesenchymal stem cells from patients with osteoporosis through the miR-30d-5p/ITGB3 axis. *Tissue Cell* 76, 101793. doi:10.1016/j.tice.2022.101793

Tang, J. Z., Zhao, G. Y., Zhao, J. Z., Di, D. H., Wang, B. (2021). lncRNA IGF2-AS promotes the osteogenic differentiation of bone marrow mesenchymal stem cells by sponging miR-3,126-5p to upregulate KLK4. *J. Gene Med.* 23 (10), e3372. doi:10.1002/jgm.3372

Tang, L., Lu, W., Huang, J., Tang, X., Zhang, H., and Liu, S. (2019). miR‑144 promotes the proliferation and differentiation of bone mesenchymal stem cells by downregulating the expression of SFRP1. *Mol. Med. Rep.* 20 (1), 270-280. doi:10.3892/mmr.2019.10252

Tang, L., Yin, Y., Liu, J., Li, Z., and Lu, X. (2017). MiR-124 attenuates osteoclastogenic differentiation of bone marrow monocytes via targeting Rab27a. *Cell. Physiol. Biochem.* 43 (4), 1663-1672. doi:10.1159/000484027

Tang, X., Lin, J., Wang, G., and Lu, J. (2017). MicroRNA-433-3p promotes osteoblast differentiation through targeting DKK1 expression. *PLoS One* 12 (6), e0179860. doi:10.1371/journal.pone.0179860

Tong, J., Zhang, M., Li, X., and Ren, G. (2021). MicroRNA‑338‑3p regulates age‑associated osteoporosis via targeting PCSK5. *Mol. Med. Rep*. 23 (2), 136. doi:10.3892/mmr.2020.11775

Visconti, V. V., Fittipaldi, S., Ciuffi, S., Marini, F., Isaia, G., D'Amelio, P., et al. (2020). Circulating long non-coding RNA GAS5 is overexpressed in serum from osteoporotic patients and is associated with increased risk of bone fragility. *Int. J. Mol. Sci.* 21 (18), 6930. doi:10.3390/ijms21186930

Wang, C. G., Hu, Y. H., Su, S. L., and Zhong, D. (2020). LncRNA DANCR and miR-320a suppressed osteogenic differentiation in osteoporosis by directly inhibiting the Wnt/β-catenin signaling pathway. *Exp. Mol. Med.* 52 (8), 1310-1325. doi:10.1038/s12276-020-0475-0

Wang, C. G., Liao, Z., Xiao, H., Liu, H., Hu, Y. H., Liao, Q. D., et al. (2019). LncRNA KCNQ1OT1 promoted BMP2 expression to regulate osteogenic differentiation by sponging miRNA-214. *Exp. Mol. Pathol.* 107, 77-84. doi:10.1016/j.yexmp.2019.01.012

Wang, C., He, H., Wang, L., Jiang, Y., and Xu, Y. (2018). Reduced *miR-144-3p* expression in serum and bone mediates osteoporosis pathogenesis by targeting RANK. *Biochem. Cell Biol.* 96 (5), 627-635. doi:10.1139/bcb-2017-0243

Wang, C., Shan, S., Wang, C., Wang, J., Li, J., Hu, G., et al. (2017). Mechanical stimulation promote the osteogenic differentiation of bone marrow stromal cells through epigenetic regulation of Sonic Hedgehog. *Exp. Cell Res.* 352 (2), 346-356. doi:10.1016/j.yexcr.2017.02.021

Wang, C., Xie, Q., Sun, W., Zhou, Y., and Liu, Y. (2021). lncRNA WT1-AS is upregulated in osteoporosis and regulates the apoptosis of osteoblasts by interacting with p53. *Exp. Ther. Med.* 22 (1), 734. doi:10.3892/etm.2021.10166

Wang, F., Deng, H., Chen, J., Wang, Z., and Yin, R. (2022). LncRNA MIAT can regulate the proliferation, apoptosis, and osteogenic differentiation of bone marrow-derived mesenchymal stem cells by targeting miR-150-5p. *Bioengineered* 13 (3), 6343-6352. doi:10.1080/21655979.2021.2011632

Wang, F. R., Zhang, F., and Zheng, F. (2022). lncRNA Kcnq1ot1 promotes bone formation by inhibiting miR-98-5p/Tbx5 axis in MC3T3-E1 cells. *Exp. Ther. Med.* 23 (3), 194. doi:10.3892/etm.2022.11117

Wang, G., Wan, L., Zhang, L., Yan, C., and Zhang, Y. (2021a). MicroRNA-133a regulates the viability and differentiation fate of bone marrow mesenchymal stem cells via MAPK/ERK signaling pathway by targeting FGFR1. *DNA Cell Biol*. 40 (8), 1112-1123. doi:10.1089/dna.2021.0206

Wang, G., Wang, F., Zhang, L., Yan, C., and Zhang, Y. (2021b). miR-133a silencing rescues glucocorticoid-induced bone loss by regulating the MAPK/ERK signaling pathway. *Stem Cell Res. Ther.* 12 (1), 215. doi:10.1186/s13287-021-02278-w

Wang, H., Li, Y. K., Cui, M., Liu, L. H., Zhao, L. M., and Wang, X. M. (2020). Effect of lncRNA AK125437 on postmenopausal osteoporosis rats via MAPK pathway. *Eur. Rev. Med. Pharmacol. Sci.* 24 (5), 2173-2180. doi:10.26355/eurrev_202003_20482

Wang, H., Shi, X., Guo, Z., Zhao, F., He, W., Kang, M., et al. (2022). microRNA-211-5p predicts the progression of postmenopausal osteoporosis and attenuates osteogenesis by targeting dual specific phosphatase 6. *Bioengineered* 13 (3), 5709-5723. doi:10.1080/21655979.2021.2017626

Wang, H., Zhao, W., Tian, Q. J., Xin, L., Cui, M., and Li, Y. K. (2020). Effect of lncRNA AK023948 on rats with postmenopausal osteoporosis via PI3K/AKT signaling pathway. *Eur. Rev. Med. Pharmacol. Sci.* 24 (5), 2181-2188. doi:10.26355/eurrev_202003_20483

Wang, J., Fu, Q., Yang, J., Liu, J. L., Hou, S. M., Huang, X., et al. (2021). RNA N6-methyladenosine demethylase FTO promotes osteoporosis through demethylating Runx2 mRNA and inhibiting osteogenic differentiation. *Aging (Albany NY)* 13 (17), 21134-21141. doi:10.18632/aging.203377

Wang, J., Gao, Z., and Gao, P. (2021). MiR-133b modulates the osteoblast differentiation to prevent osteoporosis via targeting GNB4. *Biochem. Genet.* 59 (5), 1146-1157. doi:10.1007/s10528-021-10048-9

Wang, L., Niu, N., Li, L., Shao, R., Ouyang, H., and Zou, W. (2018). H3K36 trimethylation mediated by SETD2 regulates the fate of bone marrow mesenchymal stem cells. *PLoS Biol.* 16 (11), e2006522. doi:10.1371/journal.pbio.2006522

Wang, Q., Li, Y., Zhang, Y., Ma, L., Lin, L., Meng, J., et al. (2017). LncRNA MEG3 inhibited osteogenic differentiation of bone marrow mesenchymal stem cells from postmenopausal osteoporosis by targeting miR-133a-3p. *Biomed. Pharmacother.* 89, 1178-1186. doi:10.1016/j.biopha.2017.02.090

Wang, Q., Wang, C. H., and Meng, Y. (2019). microRNA-1297 promotes the progression of osteoporosis through regulation of osteogenesis of bone marrow mesenchymal stem cells by targeting WNT5A. *Eur. Rev. Med. Pharmacol. Sci.* 23 (11), 4541-4550

Wang, T., Zhang, C., Wu, C., Liu, J., Yu, H., Zhou, X., et al. (2020). miR-765 inhibits the osteogenic differentiation of human bone marrow mesenchymal stem cells by targeting BMP6 via regulating the BMP6/Smad1/5/9 signaling pathway. *Stem Cell Res. Ther.* 11 (1), 62. doi:10.1186/s13287-020-1579-0

Wang, W. W., Yang, L., Wu, J., Gao, C., Zhu, Y. X., Zhang, D., et al. (2017). The function of miR-218 and miR-618 in postmenopausal osteoporosis. *Eur. Rev. Med. Pharmacol. Sci.* 21 (24), 5534-5541. doi:10.26355/eurrev_201712_13989

Wang, W., Li, T., and Feng, S. (2021). Knockdown of long non-coding RNA HOTAIR promotes bone marrow mesenchymal stem cell differentiation by sponging microRNA miR-378g that inhibits nicotinamide N-methyltransferase. *Bioengineered* 12 (2), 12482-12497. doi:10.1080/21655979.2021.2006863

Wang, W., Yang, L., Zhang, D., Gao, C., Wu, J., Zhu, Y., et al. (2018). MicroRNA-218 negatively regulates osteoclastogenic differentiation by repressing the nuclear factor-κB signaling pathway and targeting tumor necrosis factor receptor 1. *Cell. Physiol. Biochem.* 48 (1), 339-347. doi:10.1159/000491740

Wang, X. B., Li, P. B., Guo, S. F., Yang, Q. S., Chen, Z. X., Wang, D., et al. (2019). circRNA_0006393 promotes osteogenesis in glucocorticoid‑induced osteoporosis by sponging miR‑145‑5p and upregulating FOXO1. *Mol. Med. Rep.* 20 (3), 2851-2858. doi:10.3892/mmr.2019.10497

Wang, X. J., Liu, J. W., and Liu, J. (2020). MiR-655-3p inhibits the progression of osteoporosis by targeting LSD1 and activating BMP-2/Smad signaling pathway. *Hum. Exp. Toxicol.* 39 (10), 1390-1404. doi:10.1177/0960327120924080

Wang, X., Chen, T., Deng, Z., Gao, W., Liang, T., Qiu, X., et al. (2021). Melatonin promotes bone marrow mesenchymal stem cell osteogenic differentiation and prevents osteoporosis development through modulating circ_0003865 that sponges miR-3653-3p. *Stem Cell Res. Ther.* 12 (1), 150. doi:10.1186/s13287-021-02224-w

Wang, X., Guo, B., Li, Q., Peng, J., Yang, Z., Wang, A., et al. (2013). miR-214 targets *ATF4* to inhibit bone formation. *Nat. Med.* 19(1), 93-100. doi:10.1038/nm.3026

Wang, X., Mi, Y., He, W., Hu, X., Yang, S., Zhao, L., et al. (2021). Z Down-regulation of miR-340-5p promoted osteogenic differentiation through regulation of runt-related transcription factor-2 (RUNX2) in MC3T3-E1 cells. *Bioengineered* 12 (1), 1126-1137. doi:10.1080/21655979.2021.1905259

Wang, X., Zhao, D., Zhu, Y., Dong, Y., and Liu, Y. (2019). Long non-coding RNA GAS5 promotes osteogenic differentiation of bone marrow mesenchymal stem cells by regulating the miR-135a-5p/FOXO1 pathway. *Mol. Cell. Endocrinol.* 496, 110534. doi:10.1016/j.mce.2019.110534

Wang, Y., Deng, P., Liu, Y., Wu, Y., Chen, Y., Guo, Y., et al. (2020). Alpha-ketoglutarate ameliorates age-related osteoporosis via regulating histone methylations. *Nat. Commun.* 11 (1), 5596. doi:10.1038/s41467-020-19360-1

Wang, Y., Liu, H., Wu, J., Liao, Y., Lu, D., Dong, F., et al. (2020). 5-Aza-2-deoxycytidine inhibits osteolysis induced by titanium particles by regulating RANKL/OPG ratio. *Biochem. Biophys. Res. Commun.* 529 (3), 629-634. doi:10.1016/j.bbrc.2020.05.192

Wang, Y., Wang, K., Hu, Z., Zhou, H., Zhang, L., Wang, H., et al. (2018). MicroRNA-139-3p regulates osteoblast differentiation and apoptosis by targeting ELK1 and interacting with long noncoding RNA ODSM. *Cell Death Dis.* 9 (11), 1107. doi:10.1038/s41419-018-1153-1

Wen, J., Guan, Z., Yu, B., Guo, J., Shi, Y., and Hu, L. (2020). Circular RNA hsa_circ_0076906 competes with OGN for miR-1305 biding site to alleviate the progression of osteoporosis. *Int. J. Biochem. Cell Biol.* 122, 105719. doi:10.1016/j.biocel.2020.105719

Weng, W., Di, S., Xing, S., Sun, Z., Shen, Z., Dou, X., et al. (2021). Long non-coding RNA DANCR modulates osteogenic differentiation by regulating the miR-1301-3p/PROX1 axis. *Mol. Cell. Biochem.* 476 (6), 2503-2512. doi:10.1007/s11010-021-04074-9

Wu, J. C., Sun, J., Xu, J. C., Zhou, Z. Y., Zhang, Y. F. (2021). Down-regulated microRNA-199a-3p enhances osteogenic differentiation of bone marrow mesenchymal stem cells by targeting Kdm3a in ovariectomized rats. *Biochem. J.* 478 (4), 721-734. doi:10.1042/BCJ20200314

Wu, J., Lin, T., Gao, Y., Li, X., Yang, C., Zhang, K., et al. (2022). Long noncoding RNA *ZFAS1* suppresses osteogenic differentiation of bone marrow-derived mesenchymal stem cells by upregulating miR-499-EPHA5 axis. *Mol. Cell. Endocrinol.* 539, 111490. doi:10.1016/j.mce.2021.111490

Wu, M., Dai, M., Liu, X., Zeng, Q., and Lu, Y. (2023). lncRNA SERPINB9P1 regulates SIRT6 mediated osteogenic differentiation of BMSCs via miR-545-3p. *Calcif. Tissue Int.* 112 (1), 92-102. doi:10.1007/s00223-022-01034-3

Wu, R. W., Lian, W. S., Chen, Y. S., Kuo, C. W., Ke, H. C., Hsieh, C. K., et al. (2019). MicroRNA-29a counteracts glucocorticoid induction of bone loss through repressing TNFSF13b modulation of osteoclastogenesis. *Int. J. Mol. Sci*. 20 (20), 5141. doi:10.3390/ijms20205141

Wu, Y., Xie, L., Wang, M., Xiong, Q., Guo, Y., Liang, Y., et al. (2018). Mettl3-mediated m6A RNA methylation regulates the fate of bone marrow mesenchymal stem cells and osteoporosis. *Nat. Commun.* 9 (1), 4772. doi:10.1038/s41467-018-06898-4

Xia, Z. L., Wang, Y., Sun, Q. D., and Du, X. F. (2018). MiR-203 is involved in osteoporosis by regulating DKK1 and inhibiting osteogenic differentiation of MSCs. *Eur. Rev. Med. Pharmacol. Sci.* 2018, 22 (16), 5098-5105. doi:10.26355/eurrev_201808_15703

Xiao, J., Qin, S., Li, W., Yao, L., Huang, P., Liao, J., et al. (2020). Osteogenic differentiation of rat bone mesenchymal stem cells modulated by MiR-186 via SIRT6. *Life Sci.* 253, 117660. doi:10.1016/j.lfs.2020.117660

Xiao, Y., Li, B., and Liu, J. (2018). MicroRNA‑148a inhibition protects against ovariectomy‑induced osteoporosis through PI3K/AKT signaling by estrogen receptor α. *Mol. Med. Rep.* 17 (6), 7789-7796. doi:10.3892/mmr.2018.8845

Xie, H., Cao, L., Ye, L., Shan, G., and Song, W. (2021). The miR-1906 mimic attenuates bone loss in osteoporosis by down-regulating the TLR4/MyD88/NF-κB pathway. *Physiol. Int.* 107 (4), 469-478. doi:10.1556/2060.2020.00042

Xie, Y., Han, N., Li, F., Wang, L., Liu, G., Hu, M., et al. (2022). Melatonin enhances osteoblastogenesis of senescent bone marrow stromal cells through NSD2-mediated chromatin remodelling. *Clin. Transl. Med.* 12 (2), e746. doi:10.1002/ctm2.746

Xu, A., Yang, Y., Shao, Y., Wu, M., and Sun, Y. (2019). Inhibiting effect of microRNA-187-3p on osteogenic differentiation of osteoblast precursor cells by suppressing cannabinoid receptor type 2. *Differentiation* 109, 9-15. doi:10.1016/j.diff.2019.07.002

Xu, D., Gao, Y., Hu, N., Wu, L., and Chen, Q. (2017). miR-365 ameliorates dexamethasone-induced suppression of osteogenesis in MC3T3-E1 cells by targeting HDAC4. *Int. J. Mol. Sci.* 18 (5), 977. doi:10.3390/ijms18050977

Xu, R., Shen, X., Xie, H., Zhang, H., Liu, D., Chen, X., et al. (2021). Identification of the canonical and noncanonical role of miR-143/145 in estrogen-deficient bone loss. *Theranostics* 11 (11), 5491-5510. doi:10.7150/thno.55041

Xu, T., Zhou, P., Li, H., Ding, Q., and Hua, F. (2022). MicroRNA-577 aggravates bone loss and bone remodeling by targeting thyroid stimulating hormone receptor in hyperthyroid-associated osteoporosis. *Environ. Toxicol.* 37 (3), 539-548. doi:10.1002/tox.23419

Xu, Y., Ma, J., Xu, G., and Ma, D. (2021a). Recent advances in the epigenetics of bone metabolism. *J. Bone Miner. Metab.* 39 (6), 914-924. doi:10.1007/s00774-021-01249-8

Xu, Y., Li, D., Zhu, Z., Li, L., Jin, Y., Ma, C., et al. (2020). miR‑27a‑3p negatively regulates osteogenic differentiation of MC3T3‑E1 preosteoblasts by targeting osterix. *Mol. Med. Rep.* 22 (3), 1717-1726. doi:10.3892/mmr.2020.11246

Xu, Y., Sun, L., Hu, J., and Xu, S. (2021). Knockdown of hsa_circ_0001275 reverses dexamethasone-induced osteoblast growth inhibition via mediation of miR-377/CDKN1B axis. *PLoS One* 16 (5), e0252126. doi:10.1371/journal.pone.0252126

Xu, Y. Y., Xin, R., Sun, H., Long, D., Li, Z., Liao, H., et al. (2021). Long non-coding RNAs LOC100126784 and POM121L9P derived from bone marrow mesenchymal stem cells enhance osteogenic differentiation via the miR-503-5p/SORBS1 axis. *Front. Cell Dev. Biol.* 9, 723759. doi:10.3389/fcell.2021.723759

Xu, Y., Zhang, S., Fu, D., and Lu, D. (2020). Circulating miR-374b-5p negatively regulates osteoblast differentiation in the progression of osteoporosis via targeting Wnt3 AND Runx2. *J. Biol. Regul. Homeost. Agents* 34 (2), 345-355. doi:10.23812/19-507-A-9

Yang, B. C., Kuang, M. J., Kang, J. Y., Zhao, J., Ma, J. X., and Ma, X. L. (2020). Human umbilical cord mesenchymal stem cell-derived exosomes act via the miR-1263/Mob1/Hippo signaling pathway to prevent apoptosis in disuse osteoporosis. *Biochem. Biophys. Res. Commun.* 524(4), 883-889. doi:10.1016/j.bbrc.2020.02.001

Yang, J. J., Peng, W. X., and Zhang, M. B. (2022). LncRNA KCNQ1OT1 promotes osteogenic differentiation via miR-205-5p/RICTOR axis. *Exp. Cell Res.* 415 (1), 113119. doi:10.1016/j.yexcr.2022.113119

Yang, K., Tian, N., Liu, H., Tao, X. Z., Wang, M. X., and Huang, W. (2019). LncRNAp21 promotes osteogenic differentiation of mesenchymal stem cells in the rat model of osteoporosis by the Wnt/β-catenin signaling pathway. *Eur. Rev. Med. Pharmacol. Sci.* 23 (10), 4303-4309. doi:10.26355/eurrev_201905_17935

Yang, L., Ge, D., Cao, X., Ge, Y., Chen, H., Wang, W., et al. (2016). MiR-214 attenuates osteogenic differentiation of mesenchymal stem cells via targeting FGFR1. *Cell. Physiol. Biochem.* 38 (2), 809-820. doi:10.1159/000443036

Yang, R., Chen, J., Zhang, J., Qin, R., Wang, R., Qiu, Y., et al. (2020). 1,25-Dihydroxyvitamin D protects against age-related osteoporosis by a novel VDR-Ezh2-p16 signal axis. *Aging Cell* 19 (2), e13095. doi:10.1111/acel.13095

Yang, X. M., Song, Y. Q., Li, L., Liu, D. M., and Chen, G. D. (2021). miR-1249-5p regulates the osteogenic differentiation of ADSCs by targeting PDX1. *J. Orthop. Surg. Res.* 16 (1), 10. doi:10.1186/s13018-020-02147-x

Yang, X., Wang, G., Wang, Y., Zhou, J., Yuan, H., Li, X., et al. (2019). Histone demethylase KDM7A reciprocally regulates adipogenic and osteogenic differentiation via regulation of C/EBPα and canonical Wnt signalling. *J. Cell Mol. Med.* 23 (3), 2149-2162. doi:10.1111/jcmm.14126

Yang, X., Yang, J., Lei, P., and Wen, T. (2019). LncRNA MALAT1 shuttled by bone marrow-derived mesenchymal stem cells-secreted exosomes alleviates osteoporosis through mediating microRNA-34c/SATB2 axis. *Aging (Albany NY)* 11 (20), 8777-8791. doi:10.18632/aging.102264

Ye, L. C., Qian, L. F., Liang, L., Jiang, L. J., Che, Z. Y., Guo, Y. H. (2021). Overexpression of miR-195-5p reduces osteoporosis through activating BMP-2/SMAD/AKT/RUNX2 pathway via targeting SMURF1. *J. Biol. Regul. Homeost. Agents* 35(4). doi:10.23812/21-162-A

Ye, L., Fan, Z., Yu, B., Chang, J., Al Hezaimi, K., Zhou, X., et al. (2012). Histone demethylases KDM4B and KDM6B promotes osteogenic differentiation of human MSCs. *Cell Stem Cell* 11 (1), 50-61. doi:10.1016/j.stem.2012.04.009

Yin, C., Tian, Y., Yu, Y., Li, D., Miao, Z., Su, P., et al. (2021). Long noncoding RNA AK039312 and AK079370 inhibits bone formation via miR-199b-5p. *Pharmacol. Res.* 163, 105230. doi:10.1016/j.phrs.2020.105230

Yin, C., Tian, Y., Yu, Y., Wang, H., Wu, Z., Huang, Z., et al. (2019). A novel long noncoding RNA AK016739 inhibits osteoblast differentiation and bone formation. *J. Cell. Physiol.* 234 (7), 11524-11536. doi:10.1002/jcp.27815

Yin, J., Xiao, W., Zhao, Q., Sun, J., Zhou, W., and Zhao, W. (2022). MicroRNA-582-3p regulates osteoporosis through regulating homeobox A10 and osteoblast differentiation. *Immunopharmacol. Immunotoxicol.* 44 (3), 421-428. doi:10.1080/08923973.2022.2052895

Yin, N., Zhu, L., Ding, L., Yuan, J., Du, L., Pan, M., et al. (2019). MiR-135-5p promotes osteoblast differentiation by targeting HIF1AN in MC3T3-E1 cells. *Cell Mol. Biol. Lett.* 24, 51. doi:10.1186/s11658-019-0177-6

Yin, Z., Shen, J., Wang, Q., Wen, L., Qu, W., and Zhang, Y. (2022). miR-215-5p regulates osteoporosis development and osteogenic differentiation by targeting XIAP. *BMC Musculoskelet. Disord.* 23(1), 789. doi:10.1186/s12891-022-05731-w

You, L., Gu, W., Chen, L., Pan, L., Chen, J., and Peng, Y. (2014). MiR-378 overexpression attenuates high glucose-suppressed osteogenic differentiation through targeting CASP3 and activating PI3K/AKT signaling pathway. *Int. J. Clin. Exp. Pathol.* 7 (10), 7249-7261.

You, L., Pan, L., Chen, L., Gu, W., and Chen, J. (2016). MiR-27a is essential for the shift from osteogenic differentiation to adipogenic differentiation of Mesenchymal stem cells in postmenopausal osteoporosis. *Cell. Physiol. Biochem.* 39 (1), 253-265. doi:10.1159/000445621

You, M., Zhang, L., Zhang, X., Fu, Y., and Dong, X. (2021). MicroRNA-197-3p inhibits the osteogenic differentiation in osteoporosis by down-regulating KLF 10. *Clin. Interv. Aging* 16, 107-117. doi:10.2147/CIA.S269171

Yu, H., Li, Y., Tang, J., Lu, X., Hu, W., and Cheng, L. (2021). Long non-coding RNA RP11-84C13.1 promotes osteogenic differentiation of bone mesenchymal stem cells and alleviates osteoporosis progression via the miR-23b-3p/RUNX2 axis. *Exp. Ther. Med.* 22 (5), 1340. doi:10.3892/etm.2021.10775

Yu, J., Xiao, M., and Ren, G. (2021). Long non-coding RNA XIST promotes osteoporosis by inhibiting the differentiation of bone marrow mesenchymal stem cell by sponging miR-29b-3p that suppresses nicotinamide N-methyltransferase. *Bioengineered* 12 (1), 6057-6069. doi:10.1080/21655979.2021.1967711

Yu, T., You, X., Zhou, H., He, W., Li, Z., Li, B., et al. (2020). MiR-16-5p regulates postmenopausal osteoporosis by directly targeting VEGFA. *Aging (Albany NY)* 12 (10), 9500-9514. doi:10.18632/aging.103223

Yu, X., Rong, P. Z., Song, M. S., Shi, Z. W., Feng, G., Chen, X. J., et al. (2021). lncRNA SNHG1 induced by SP1 regulates bone remodeling and angiogenesis via sponging miR-181c-5p and modulating SFRP1/Wnt signaling pathway. *Mol. Med.* 27 (1), 141. doi:10.1186/s10020-021-00392-2

Yu, X., Song, M. S., Rong, P. Z., Chen, X. J., Shi, L., Wang, C. H., et al. (2022). LncRNA SNHG1 modulates adipogenic differentiation of BMSCs by promoting DNMT1 mediated Opg hypermethylation via interacting with PTBP1. *J. Cell. Mol. Med.* 26 (1), 60-74. doi:10.1111/jcmm.16982

Yu, Y., Cai, W., Xu, Y., and Zuo, W. (2022). Down-regulation of miR-19b-3p enhances IGF-1 expression to induce osteoblast differentiation and improve osteoporosis. *Cell. Mol. Biol. (Noisy-le-grand)* 68 (1), 160-168. doi:10.14715/cmb/2022.68.1.20

Yuan, F., Chen, H., Hu, P., Su, P., and Guan, X. (2021). MiR-26a regulates the expression of serum IGF-1 in patients with osteoporosis and its effect on proliferation and apoptosis of mouse chondrocytes. *J. Musculoskelet. Neuronal. Interact.* 21 (2), 298-307

Zha, J. P., Wang, X. Q., and Di, J. (2020). MiR-920 promotes osteogenic differentiation of human bone mesenchymal stem cells by targeting HOXA7. *J. Orthop. Surg. Res.* 15 (1), 254. doi:10.1186/s13018-020-01775-7

Zhang, D. W., Wang, H. G., Zhang, K. B., Guo, Y. Q., Yang, L. J., and Lv, H. (2022). LncRNA XIST facilitates S1P-mediated osteoclast differentiation via interacting with FUS. *J. Bone Miner. Metab.* 40(2):240-250. doi:10.1007/s00774-021-01294-3

Zhang, H. G., Wang, X. B., Zhao, H., and Zhou, C. N. (2019). MicroRNA-9-5p promotes osteoporosis development through inhibiting osteogenesis and promoting adipogenesis via targeting Wnt3a. *Eur. Rev. Med. Pharmacol. Sci.* 23 (2), 456-463. doi:10.26355/eurrev_201901_16855

Zhang, H. L., Du, X. Y., and Dong, Q. R. (2019). LncRNA XIXT promotes osteogenic differentiation of bone mesenchymal stem cells and alleviates osteoporosis progression by targeting miRNA-30a-5p. *Eur. Rev. Med. Pharmacol. Sci.* 23 (20), 8721-8729. doi:10.26355/eurrev_201910_19266

Zhang, H., Chen, L., Wang, Z., Sun, Z., Shan, Y., Li, Q., et al. (2022). Long noncoding RNA KCNQ1OT1 inhibits osteoclast differentiation by regulating the miR-128-3p/NFAT5 axis. *Aging (Albany NY)* 14 (10), 4486-4499. doi:10.18632/aging.204088

Zhang, J., Zhang, T., Tang, B., Li, J., and Zha, Z. (2021). The miR-187 induced bone reconstruction and healing in a mouse model of osteoporosis, and accelerated osteoblastic differentiation of human multipotent stromal cells by targeting BARX2. *Pathol. Res. Pract.* 219, 153340. doi:10.1016/j.prp.2021.153340

Zhang, L., Li, S., Li, J., and Li, Y. (2021). LncRNA ORLNC1 promotes bone marrow mesenchyml stem cell pyroptosis induced by advanced glycation end production by targeting miR-200b-3p/Foxo3 pathway. *Stem Cell Rev. Rep.* 17 (6), 2262-2275. doi:10.1007/s12015-021-10247-2

Zhang, L., Xie, H., and Li, S. (2020). LncRNA LOXL1-AS1 controls osteogenic and adipocytic differentiation of bone marrow mesenchymal stem cells in postmenopausal osteoporosis through regulating the miR-196a-5p/Hmga2 axis. *J. Bone Miner. Metab.* 38 (6), 794-805. doi:10.1007/s00774-020-01123-z

Zhang, P., Liu, Y., Jin, C., Zhang, M., Lv, L., Zhang, X., et al. (2016). Histone H3K9 acetyltransferase PCAF is essential for osteogenic differentiation through bone morphogenetic protein signaling and may be involved in osteoporosis. *Stem Cells* 34 (9), 2332-2341. doi:10.1002/stem.2424

Zhang, R. F., Liu, J. W., Yu, S. P., Sun, D., Wang, X. H., Fu, J. S., et al. (2019). LncRNA UCA1 affects osteoblast proliferation and differentiation by regulating BMP-2 expression. *Eur. Rev. Med. Pharmacol. Sci.* 23 (16), 6774-6782. doi:10.26355/eurrev_201908_18715

Zhang, S. Y., Gao, F., Peng, C. G., Zheng, C. J., and Wu, M. F. (2018). miR-485-5p promotes osteoporosis via targeting Osterix. *Eur. Rev. Med. Pharmacol. Sci.* 22 (15), 4792-4799. doi:10.26355/eurrev_201808_15613

Zhang, S., Wu, W., Jiao, G., Li, C., and Liu, H. (2018). MiR-455-3p activates Nrf2/ARE signaling via HDAC2 and protects osteoblasts from oxidative stress. Int J Biol Macromol. 107 (Pt B), 2094-2101. doi:10.1016/j.ijbiomac.2017.10.080

Zhang, W., Wu, Y., Shiozaki, Y., Sugimoto, Y., Takigawa, T., Tanaka, M., et al. (2018). miRNA-133a-5p inhibits the expression of osteoblast differentiation-associated markers by targeting the 3' UTR of RUNX2. *DNA Cell Biol.* 37 (3), 199-209. doi:10.1089/dna.2017.3936

Zhang, X. H., Geng, G. L., Su, B., Liang, C. P., Wang, F., and Bao, J. C. (2016). MicroRNA-338-3p inhibits glucocorticoid-induced osteoclast formation through RANKL targeting. *Genet. Mol. Res.* 15 (3). doi:10.4238/gmr.15037674

Zhang, X., Zhu, Y., Zhang, C., Liu, J., Sun, T., Li, D., et al. (2018). miR-542-3p prevents ovariectomy-induced osteoporosis in rats via targeting SFRP1. *J. Cell. Physiol.* 233 (9), 6798-6806. doi:10.1002/jcp.26430

Zhang, Y. X., Sun, H. L., Liang, H., Li, K., Fan, Q. M., and Zhao, Q. H. (2015). Dynamic and distinct histone modifications of osteogenic genes during osteogenic differentiation. *J. Biochem.* 158 (6), 445-457. doi:10.1093/jb/mvv059

Zhang, Y., Cao, X., Li, P., Fan, Y., Zhang, L., Ma, X., et al. (2021). microRNA-935-modified bone marrow mesenchymal stem cells-derived exosomes enhance osteoblast proliferation and differentiation in osteoporotic rats. *Life Sci.* 272, 119204. doi:10.1016/j.lfs.2021.119204

Zhang, Y., Chen, B., Li, D., Zhou, X., and Chen, Z. (2019). LncRNA NEAT1/miR-29b-3p/BMP1 axis promotes osteogenic differentiation in human bone marrow-derived mesenchymal stem cells. *Pathol. Res. Pract.* 215 (3), 525-531. doi:10.1016/j.prp.2018.12.034

Zhang, Y., Gao, Y., Cai, L., Li, F., Lou, Y., Xu, N., et al. (2017). MicroRNA-221 is involved in the regulation of osteoporosis through regulates RUNX2 protein expression and osteoblast differentiation. *Am. J. Transl. Res.* 9 (1), 126-135

Zhang, Y., Jiang, Y., Luo, Y., and Zeng, Y. (2020). Interference of miR-212 and miR-384 promotes osteogenic differentiation via targeting RUNX2 in osteoporosis. *Exp. Mol. Pathol.* 113, 104366. doi:10.1016/j.yexmp.2019.104366

Zhang, Y., Liu, Y., Wu, M., Wang, H., Wu, L., Xu, B., et al. (2020). MicroRNA-664a-5p promotes osteogenic differentiation of human bone marrow-derived mesenchymal stem cells by directly downregulating HMGA2. *Biochem. Biophys. Res. Commun.* 521 (1), 9-14. doi:10.1016/j.bbrc.2019.09.122

Zhang, Y., Ma, C., Liu, X., Wu, Z., Yan, P., Ma, N., et al. (2015). Epigenetic landscape in PPARγ2 in the enhancement of adipogenesis of mouse osteoporotic bone marrow stromal cell. *Biochim. Biophys. Acta* 1852 (11), 2504-2516. doi:10.1016/j.bbadis.2015.08.020

Zhang, Z., Jiang, W., Hu, M., Gao, R., and Zhou, X. (2021). MiR-486-3p promotes osteogenic differentiation of BMSC by targeting CTNNBIP1 and activating the Wnt/β-catenin pathway. *Biochem. Biophys. Res. Commun.* 566, 59-66. doi:10.1016/j.bbrc.2021.05.098

Zhao, C., Sun, W., Zhang, P., Ling, S., Li, Y., Zhao, D., et al. (2015). miR-214 promotes osteoclastogenesis by targeting Pten/PI3k/AKT pathway. *RNA Biol.* 12 (3), 343-53. doi:10.1080/15476286.2015.1017205

Zhao, F., Xu, Y., Ouyang, Y., Wen, Z., Zheng, G., Wan, T., et al. (2021). Silencing of miR-483-5p alleviates postmenopausal osteoporosis by targeting SATB2 and PI3K/AKT pathway. *Aging (Albany NY)* 13 (5), 6945-6956. doi:10.18632/aging.202552

Zhao, G., Luo, W. D., Yuan, Y., Lin, F., Guo, L. M., Ma, J. J., et al. (2022). LINC02381, a sponge of miR-21, weakens osteogenic differentiation of hUC-MSCs through KLF12-mediated Wnt4 transcriptional repression. *J. Bone Miner. Metab.* 40 (1), 66-80. doi:10.1007/s00774-021-01277-4

Zhao, H., Yang, Y., Wang, Y., Feng, X., Deng, A., Ou, Z., et al. (2020). MicroRNA-497-5p stimulates osteoblast differentiation through HMGA2-mediated JNK signaling pathway. *J. Orthop. Surg. Res.* 15 (1), 515. doi:10.1186/s13018-020-02043-4

Zhao, J., Huang, M., Zhang, X., Xu, J., Hu, G., Zhao, X., et al. (2019). MiR-146a deletion protects from bone loss in OVX mice by suppressing RANKL/OPG and M-CSF in bone microenvironment. *J. Bone Miner. Res*. 34 (11), 2149-2161. doi:10.1002/jbmr.3832

Zhao, J., Liu, S., Zhang, W., Ni, L., Hu, Z., Sheng, Z., et al. (2019). MiR-128 inhibits the osteogenic differentiation in osteoporosis by down-regulating SIRT6 expression. *Biosci. Rep.* 39 (9), BSR20191405. doi:10.1042/BSR20191405

Zhao, M., Dong, J., Liao, Y., Lu, G., Pan, W., Zhou, H., et al. (2022). MicroRNA miR-18a-3p promotes osteoporosis and possibly contributes to spinal fracture by inhibiting the glutamate AMPA receptor subunit 1 gene (GRIA1). *Bioengineered* 13 (1), 370-382. doi:10.1080/21655979.2021.2005743

Zhao, Q. H., Wang, S. G., Liu, S. X., Li, J. P., Zhang, Y. X., Sun, Z. Y., et al. (2013). PPARγ forms a bridge between DNA methylation and histone acetylation at the C/EBPα gene promoter to regulate the balance between osteogenesis and adipogenesis of bone marrow stromal cells. *FEBS J*. 280 (22), 5801-5814. doi:10.1111/febs.12500

Zhao, Y., Chen, Y., Hu, X., Zhang, N., and Wang, F. (2020). lncRNA LINC01535 upregulates BMP2 expression levels to promote osteogenic differentiation via sponging miR‑3619‑5p. *Mol. Med. Rep.* 22 (6), 5428-5435. doi:10.3892/mmr.2020.11635

Zheng, F., Zhang, F., and Wang, F. (2022). Inhibition of miR‑98‑5p promotes high glucose‑induced suppression of preosteoblast proliferation and differentiation via the activation of the PI3K/AKT/GSK3β signaling pathway by targeting BMP2. *Mol. Med. Rep.* 26 (3), 292. doi:10.3892/mmr.2022.12808

Zheng, H. B., Wu, M., Zhang, G., and Chen, K. L. (2021). MicroRNA-182 inhibits osteogenic differentiation of bone marrow mesenchymal stem cells by targeting Smad1. *J. Biol. Regul. Homeost. Agents* 35 (2), 505-516. doi:10.23812/20-688-A

Zheng, S., Wang, Y. B., Yang, Y. L., Chen, B. P., Wang, C. X., Li, R. H., et al. (2019). LncRNA MALAT1 inhibits osteogenic differentiation of mesenchymal stem cells in osteoporosis rats through MAPK signaling pathway. *Eur. Rev. Med. Pharmacol. Sci*. 23 (11), 4609-4617. doi:10.26355/eurrev_201906_18038

Zhi, F., Ding, Y., Wang, R., Yang, Y., Luo, K., and Hua, F. (2021). Exosomal hsa_circ_0006859 is a potential biomarker for postmenopausal osteoporosis and enhances adipogenic versus osteogenic differentiation in human bone marrow mesenchymal stem cells by sponging miR-431-5p. *Stem Cell Res Ther.* 12 (1), 157. doi:10.1186/s13287-021-02214-y

Zhong, L. N., Zhang, Y. Z., Li, H., Fu, H. L., Lv, C. X., and Jia, X. J. (2019). Overexpressed miR-196a accelerates osteogenic differentiation in osteoporotic mice via GNAS-dependent Hedgehog signaling pathway. *J. Cell. Biochem.* 120 (12), 19422-19431. doi:10.1002/jcb.29166

Zhou, B., Peng, K., Wang, G., Chen, W., Liu, P., Chen, F., et al. (2020). miR‑483‑3p promotes the osteogenesis of human osteoblasts by targeting Dikkopf 2 (DKK2) and the Wnt signaling pathway. *Int. J. Mol. Med.* 46 (4), 1571-1581. doi:10.3892/ijmm.2020.4694

Zhou, J. G., Hua, Y., Liu, S. W., Hu, W. Q., Qian, R., and Xiong, L. (2020). MicroRNA-1286 inhibits osteogenic differentiation of mesenchymal stem cells to promote the progression of osteoporosis via regulating FZD4 expression. *Eur. Rev. Med. Pharmacol. Sci.* 24 (1), 1-10. doi:10.26355/eurrev_202001_19889

Zhou, J., Nie, H., Liu, P., Wang, Z., Yao, B., and Yang, L. (2019). Down-regulation of miR-339 promotes differentiation of BMSCs and alleviates osteoporosis by targeting DLX5. *Eur. Rev. Med. Pharmacol. Sci.* 23 (1), 29-36. doi:10.26355/eurrev_201901_16744

Zhou, L. G., Shi, P., Sun, Y. J., Liu, H. Z., Ni, J. Q., and Wang, X. (2019). MiR-214-3p delays fracture healing in rats with osteoporotic fracture through inhibiting BMP/Smad signaling pathway. *Eur. Rev. Med. Pharmacol. Sci.* 23 (2), 449-455. doi:10.26355/eurrev_201901_16854

Zhou, L., Song, H. Y., Gao, L. L., Yang, L. Y., Mu, S., and Fu, Q. (2019). MicroRNA‑100‑5p inhibits osteoclastogenesis and bone resorption by regulating fibroblast growth factor 21. *Int. J. Mol. Med.* 43 (2), 727-738. doi:10.3892/ijmm.2018.4017

Zhou, R., Miao, S., Xu, J., Sun, L., and Chen, Y. (2021). Circular RNA circ_0000020 promotes osteogenic differentiation to reduce osteoporosis via sponging microRNA miR-142-5p to up-regulate Bone Morphogenetic Protein BMP2. *Bioengineered* 12 (1), 3824-3836. doi:10.1080/21655979.2021.1949514

Zhou, Y., Qiao, H., Liu, L., Dong, P., Zhu, F., Zhang, J., et al. (2021). miR-21 regulates osteogenic and adipogenic differentiation of BMSCs by targeting PTEN. *J. Musculoskelet. Neuronal Interact.* 21 (4), 568-576

Zhu, J., Wang, H., and Liu, H. (2020). Osteoclastic miR-301-b knockout reduces ovariectomy (OVX)-induced bone loss by regulating CYDR/NF-κB signaling pathway. *Biochem. Biophys. Res. Commun.* 529 (1), 35-42. doi:10.1016/j.bbrc.2020.05.111
